# Immunogenicity and safety of an *Escherichia coli*-produced 9-valent human papillomavirus vaccine (types 6/11/16/18/31/33/45/52/58) in healthy Chinese women aged 20–45 years: a single-center, randomized, observer-blinded, positive controlled phase 2 clinical trial

**DOI:** 10.3389/fimmu.2025.1706662

**Published:** 2025-11-14

**Authors:** Mingwei Wei, Hongyan Liu, Hongyang Yu, Hongxing Pan, Hong Tao, Jing Zhang, Weiwei Han, Dan Wu, Wenjuan Wang, Jingxin Li

**Affiliations:** 1 School of Public Health, National Vaccine Innovation Platform, Nanjing Medical University, Nanjing, China; 2 Department of Vaccine Clinical Evaluation, Jiangsu Provincial Center for Disease Control and Prevention (Jiangsu Provincial Academy of Preventive Medicine), Nanjing, China; 3 Jiangsu Provincial Medical Innovation Center, National Health Commission (NHC) Key Laboratory of Enteric Pathogenic Microbiology, Jiangsu Provincial Center for Disease Control and Prevention (Jiangsu Provincial Academy of Preventive Medicine), Nanjing, China; 4 Beijing Health Guard Biotechnology Inc., Beijing, China; 5 Sheyang City Center for Disease Control and Prevention, Yancheng, China

**Keywords:** human papillomavirus, 9-valent human papillomavirus vaccine, *Escherichia coli*, immunogenicity, safety

## Abstract

**Background:**

Vaccination with prophylactic human papillomavirus (HPV) vaccines is one of the most effective measures to prevent cervical cancer and other related diseases. Here we aimed to evaluate the immunogenicity and safety of an *Escherichia coli*-produced 9-valent human papillomavirus (9vHPV) vaccine.

**Method:**

We did a single-center, randomized, observer-blinded, positive controlled phase 2 clinical trial in healthy women aged 20–45 years. All eligible participants were randomly assigned (1:1:1) to receive 270 ug 9vHPV, 360 ug 9vHPV, or the control vaccine (Gardasil) with a 0–2–6-month schedule. Serum samples were collected at day 0 and month 7 to assess IgG and neutralizing antibodies (nAbs). For HPV 6/HPV 11/HPV 16/HPV 18, non-inferiority was identified for the lower limit of the 95% CI of the geometric mean titer (GMT) ratio at a margin of 0.5 and a seroconversion rate (SCR) difference at a margin of -5%. For HPV 31/HPV 33/HPV 45/HPV 52/HPV 58, superiority was demonstrated if the lower limit of the 95% CI for GMT ratio is greater than 1.

**Results:**

A total of 780 participants aged 20–45 years were enrolled, among whom 770 completed the three-dose immunization schedule. The incidences of ARs within 7 days in the 270 μg, 360 μg, and positive control groups were 38.85%, 41.92%, and 22.69%, respectively (*P* < 0.001). The GMTs of nAbs and IgG antibodies for nine HPV types in both 270 ug and 360 ug groups showed an obvious rise at month 7. For HPV 31/HPV 33/HPV 45/HPV 52/HPV 58, the GMTs of nAbs in both 270 μg and 360 μg groups were higher than those in the positive control group, with the 95% CI lower bounds of GMT ratios all greater than 1. Compared to the positive control group, HPV 6 and HPV 18 achieved non-inferiority criteria for GMT in both dose groups. However, the GMT ratio of HPV 16 in the 270 μg group was 0.46 (0.38–0.55), and HPV 16 and HPV 11 in the 360 μg group were 0.48 (0.40–0.58) and 0.53 (0.46–0.60), respectively. The SCRs of nAbs for HPV 6/HPV 11/HPV 16/HPV 18 in the three groups were 100%, with the 95% CI lower bound for SCR differences ranging from -2.55% to -1.64%.

**Conclusion:**

The candidate 9vHPV vaccine was well tolerated and immunogenic, supporting further evaluation for efficacy and safety in larger populations.

**Clinical Trial Registration:**

https://clinicaltrials.gov, identifier NCT05694728.

## 1 Introduction

The human papillomavirus (HPV) causes premalignant and malignant lesions of the cervix, vagina, vulva, anus, penis, and oropharynx as well as genital warts, which has imposed a substantial disease burden worldwide, especially in developing countries (1, 2). Cervical cancer is the fourth most common cancer among women. It is estimated that there were approximately 660,000 new cases and 350,000 deaths globally in 2022 (3). Currently, more than 200 HPV types have been identified, among which 12 types are recognized as “high-risk type” (HR-HPV), including HPV 16, HPV 18, HPV 31, HPV 33, HPV 35, HPV 39, HPV 45, HPV 51, HPV 52, HPV 56, HPV 58, and HPV 59 (4). HPV types 16 and 18 are the most predominant HR-HPV types, contributing to approximately 70% of cervical cancer cases, 85% of head and neck cancer cases, and 87% of anal cancer cases globally (1, 5, 6). About 90% of genital warts cases are caused by low-risk HPV types 6 and 11 (5).

Vaccination with prophylactic HPV vaccines is one of the most effective measures to prevent cervical cancer and other related diseases and is recommended as a primary preventive intervention by the World Health Organization (WHO) (7). Currently, there are five first-generation HPV vaccines on the market, all containing HR-HPV types 16 and 18, including three bivalent HPV vaccines targeting HPV types 16 and 18 (Cervarix, Cecolin, and Walrinvax) and two quadrivalent HPV vaccines targeting HPV 6, HPV 11, HPV 16, and HPV 18 (Gardasil and Cervavac) (8–10). Compared with quadrivalent vaccines, the 9vHPV vaccine includes five additional HR-HPV types (HPV 31, HPV 33, HPV 45, HPV 52, and HPV 58), which has the potential to increase the overall prevention rate of cervical cancer from approximately 70% to around 90% (11). Furthermore, the estimated preventive effect of the 9vHPV vaccine against cervical intraepithelial neoplasia grade 2 or more severe lesions has increased from 45.5% to 82.3% in Europe (12). The world’s first 9vHPV (Gardasil 9; Merck Sharp & Dohme) was approved for marketing in 2014 (7). Besides that, Cecolin 9 received approval on June 4, 2025 in China, becoming the second 9vHPV vaccine globally (13). However, the current coverage rate of HPV vaccines is generally low, especially in low-income and middle-income countries. The high cost and low production supply of the 9vHPV vaccine have become major obstacles to the accessibility of vaccination (14).

Similar to the bivalent HPV vaccine Cecolin, the candidate 9vHPV vaccine is also based on an *Escherichia coli*-expressing system (9). As an efficient expression platform, *Escherichia coli* has the advantages of low culture cost, short production cycle, and easy large-scale production, which has been proven to have good safety and extremely high efficacy. It has great potential in alleviating the shortage of HPV vaccine supply and reducing costs.

The candidate 9vHPV vaccine has demonstrated good tolerability and strong immunogenicity in phase 1 clinical trial. Here we report the immunogenicity and safety results of the *Escherichia coli*-produced recombinant 9vHPV vaccine conducted among healthy women aged 20 to 45 years in China.

## 2 Methods

### 2.1 Study design and participants

We conducted a single-center, randomized, observer-blinded, positive controlled phase 2 clinical trial to evaluate the immunogenicity and safety of an *E. coli*-produced 9vHPV vaccine in healthy women aged 20–45 years. The study was conducted in Sheyang, Jiangsu, China, from May 2020 to January 2021 (**Supplementary Figure S1**). Healthy women were enrolled if they met the following inclusion criteria: aged 20–45 years, being healthy—as determined by an investigator—clinically based on medical examination and history; axillary temperature lower than 37.0°C; negative urine pregnancy test; willingness to comply with the procedure of the study; and sexually active women with child-bearing potential who agreed to practice an effective method of contraception for 28 days before vaccination and throughout the study period. The main exclusion criteria included any previous HPV vaccination; currently pregnant or lactating or with plans to be pregnant within 7 months; any known allergy or allergic to any component of the study vaccine; history of severe adverse event with vaccines; and any other condition that may—as judged by the investigator—prevent the participant from complying with protocol or providing informed consent. The full exclusion criteria are provided in the **Supplementary Materials**.

All of the participants signed written informed consent forms before enrollment. This study has been registered at ClinicalTrials. gov (NCT05694728) and approved by the Ethics Committee of the Jiangsu Provincial Center for Disease Control and Prevention (JSJK2019-A022). The study was conducted in accordance with the principles of Good Clinical Practice, the Declaration of Helsinki, and the International Conference on Harmonization of Technical Requirements for Registration of Pharmaceuticals for Human Use guidelines.

### 2.2 Randomization and masking

All eligible participants were first stratified into 20–30 years and 31–45 years. Within each age stratum, the participants were then randomly assigned to 270 ug candidate vaccine group, 360 ug candidate vaccine group, or positive control quadrivalent human papillomavirus (qHPV) vaccine group (Gardasil; Merck Sharp & Dohme) in a ratio of 1:1:1. The randomization codes were generated by an independent statistician using SAS software (version 9.4).

Due to the differences in internal packaging between the test vaccine and control vaccine, the personnel responsible for vaccine management and administration signed confidentiality agreements and did not participate in any subsequent work, including follow-up. All participants, laboratory operators who were responsible for antibody testing, and the other study investigators were masked to group allocation and vaccine codes throughout the study.

### 2.3 Procedures

The study on 9vHPV vaccine was developed by Beijing Health Guard Biotechnology Co., Ltd., under good manufacturing practice conditions. Each 0.5-mL dose of vaccine contains a specific amount of HPV L1 VLPs. The 270 ug group included 30 μg of HPV 6, 40 μg of HPV 11 and HPV 18, 60 μg of HPV 16, and 20 μg each of HPV 31, HPV 33, HPV 45, HPV 52, and HPV 58 L1 VLPs. The 360 μg group included 30 μg of HPV 6, 40 μg of HPV 11, 80 μg of HPV 16, 60 μg of HPV 18, and 30 μg each of HPV 31, HPV 33, HPV 45, HPV 52, and HPV 58 L1 VLPs. The positive control qHPV vaccine contained a total of 120 μg HPV L1 VLPs, including 20 μg of HPV 6, 40 μg of HPV 11, 40 μg of HPV 16, and 20 μg of HPV 18 L1 VLPs.

All eligible participants were randomly assigned to receive three doses of candidate or positive control vaccine intramuscularly in the upper arm deltoid muscle according to a 0–2–6-month schedule. After each dose, the participants were observed for at least 30 min to record any immediate adverse events (AEs). The investigators provided the participants with diary cards and instructed them to record solicited local and systemic AEs for 7 days and unsolicited AEs for 30 days after each vaccination. The severity of AEs was graded according to the Guidelines for Adverse Event Classification Standards for Clinical Trials of Preventive Vaccines (2019) issued by the National Medical Products Administration (NMPA) of China. Serious adverse events (SAEs) were documented throughout the study period by a combination of spontaneous participant reports and regular follow-up.

Urine samples were collected from the participants before each vaccination for pregnancy testing, and doses were administered only if the results were negative. Blood samples from all participants were collected at day 0 before vaccination, month 3 (1 month after the second vaccination), and month 7 (1 month after the third vaccination). The samples collected at day 0 and month 7 were used to evaluate the levels of IgG and neutralizing antibodies (nAb) against specific HPV types, which were performed at the National Institute for Food and Drug Control (NIFDC) of China. The nAbs were measured by using the pseudovirion-based neutralization assay (PBNA). The neutralization titers of positive samples were determined as the highest serum dilution with a percent infection inhibition higher than 50%. IgG antibodies were detected by enzyme-linked immunosorbent assay (ELISA). The IgG antibody titers of the positive samples were calculated as the highest serum dilution. For both tests, the antibody titers of seronegative samples were assigned as half of the cutoff values for the calculation of geometric mean titers (GMTs).

In addition, the production process of this product uses glutathione-S-transferase (GST) tag protein to improve the solubility of the target protein in *Escherichia coli* and enhance the expression efficiency. The tag protein is then cleaved by 3C protease, and relevant impurities are finally removed during the purification process. Therefore, it is necessary to detect GST and 3C protease-related antibodies at day 0, month 3, and month 7, and these were conducted by Beijing Health Guard Biotechnology, Inc., using the ELISA method. Details regarding antibody detection are presented in the **Supplementary Materials**.

### 2.4 Outcomes

The primary endpoint for safety was the incidence of solicited adverse reactions (ARs) within 7 days. Since HPV types 6, 11, 16, and 18 are the core target types of most currently marketed HPV vaccines and hold crucial epidemiological and clinical significance, non-inferiority analysis can be conducted to compare the core immunogenicity between the candidate vaccine and the marketed vaccine, thereby providing a basis to determine the optimal dose. Therefore, the primary endpoint for immunogenicity was the GMT of nAbs against HPV 6, HPV 11, HPV 16, and HPV 18 generated by the 9vHPV vaccine at month 7 in participants who were nAbs baseline seronegative for the corresponding HPV types.

The secondary outcomes for safety included the incidence of unsolicited AEs within 30 days and SAEs throughout the study period. As HPV types 31, 33, 45, 52, and 58 are additional types not covered by the positive control vaccine (which lacks the corresponding antigens), superiority analysis was adopted to evaluate their immunogenicity. Thus, the secondary outcome for immunogenicity was the immune response in terms of GMT of nAbs against HPV 31, HPV 33, HPV 45, HPV 52, and HPV 58 generated by the 9vHPV vaccine at month 7 in participants who were baseline nAbs seronegative for the corresponding HPV types.

Subgroup analyses were conducted on participants who were baseline seronegative for the corresponding HPV types as well as for all participants in the per-protocol set (PPS) to evaluate the nAbs and IgG antibody levels, seroconversion rates (SCRs), and geometric mean increase (GMI) at month 7. As for the sensitivity analysis, the GMTs of nAbs were evaluated on participants who were baseline IgG negative for the corresponding HPV types. Exploratory outcomes pre-specified in the protocol were the levels of GST and 3C protease-related antibodies at day 0, month 3, and month 7.

### 2.5 Statistical analysis

The sample size of this trial was mainly estimated based on the non-inferiority hypothesis of the GMTs of antibodies against the common serotypes HPV 6, HPV 11, HPV 16, and HPV 18 induced by the test vaccine and the control vaccine. The non-inferiority margin was set at 0.5. A minimum of 164 participants per group was required as calculated by the PASS 16.0 software. Considering that the baseline seropositivity rate was approximately 30% and a dropout rate of 10%, the final sample size was set to be at least 260 participants per group, with a total of 780 participants needed.

All subjects who received at least one dose of vaccine were included in the safety analysis set (SS). The full analysis set (FAS) was defined as all enrolled subjects who received at least one dose of immunization and had at least one blood sample collected for analysis. Immunogenicity analysis was mainly based on the PPS, which included participants who received all three vaccinations and donated serum samples at day 0 and month 7 within predefined time windows and with no violation of the protocol. Seroconversion was defined as a change from seronegative at baseline to seropositive at month 7 or having four times or higher increase of antibody titers at month 7 for those who are seropositive at baseline, and the geometric mean of fold increase of the antibody titers was defined as GMI.

The differences in the incidence of AEs and ARs among the three vaccine groups were analyzed using Pearson’s chi-square test or Fisher’s exact test. The difference in antibody titers across vaccine groups was evaluated by using ANOVA. The 95% CI values of the SCR were calculated using the Clopper–Pearson exact method. The GMT and GMI, with 95% confidential intervals (CIs), were calculated based on Student’s *t* distribution of the log-transformed values. Additional comparisons were made of the nAbs GMT ratios (270 ug/positive control or 360 ug/positive control) and the SCR differences (270 ug positive control or 360 ug positive control) at month 7. For HPV 6, HPV 11, HPV 16, and HPV 18, a conclusion of non-inferiority is established if the lower limit of the two-sided 95% CI for the GMT ratio is greater than 0.5. The Miettinen–Nurminen method is used to calculate the two-sided 95% CI of the SCR difference. When the SCRs of both groups are 100%, the Newcombe method is adopted. Non-inferiority is demonstrated if the lower limit of the two-sided 95% CI of the SCR difference is greater than -5%. For HPV types unique to the 9vHPV vaccine (HPV 31, HPV 33, HPV 45, HPV 52, and HPV 58), superiority is demonstrated if the lower limit of the 95% CI for the GMT ratio is greater than 1.

Data was analyzed using SAS software (version 9.4) or GraphPad Prism (version 9.5). All statistical tests were performed using two-sided tests with an alpha value of 0.05. When a significant difference across vaccine groups was found, we further performed multiple comparisons on the basis of Bonferroni-adjusted alpha (*α* = 0.017).

## 3 Results

### 3.1 Characteristics of the study participants

**Figure 1:**
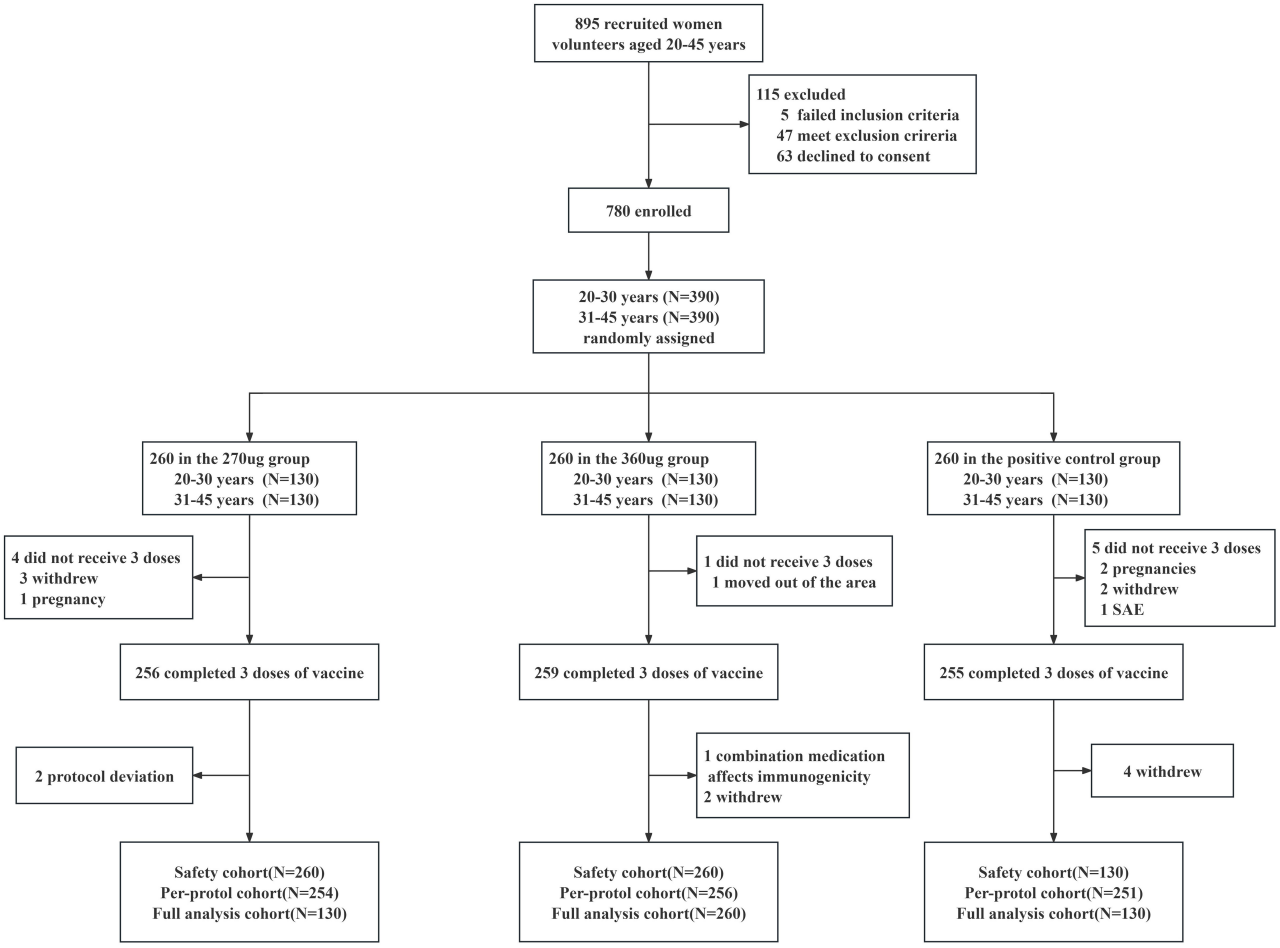
Trial profile.

From May 2020 to January 2021, a total of 895 participants aged 20–45 years underwent eligibility screening, of which five failed the inclusion criteria, 47 met the exclusion criteria, and 63 declined to give informed consent. A total of 780 healthy women were enrolled and randomly assigned (1:1:1) to 270 ug group, 360 ug group, and positive control group (**Figure 1**). Both the baseline demographic characteristics and the seropositive rates of nAbs and IgG antibodies against corresponding HPV type were generally similar across the three vaccine groups (**Table 1**).

**Table 1:** Baseline characteristics of the participants.

	270 ug (*N* = 260)	360 ug (*N* = 260)	Positive control (*N* = 260)	Total (*N* = 780)
Age (years), mean (SD)	32.43 (6.83)	32.42 (6.76)	31.78 (6.59)	32.21 (6.72)
20–30 years	26.63 (2.80)	26.66 (2.74)	26.32 (2.80)	26.54 (2.78)
31–45 years	38.22 (4.24)	38.17 (4.17)	37.24 (4.41)	37.88 (4.29)
Sex				
Male, *n* (%)	0	0	0	0
Female, *n* (%)	260 (100)	260 (100)	260 (100)	780 (100)
Height (cm), mean (SD)	159.38 (5.30)	159.23 (5.49)	159.38 (4.77)	159.33 (5.19)
Weight (kg), mean (SD)	60.38 (10.36)	61.13 (9.86)	60.49 (9.99)	60.67 (10.07)
BMI (kg/m^2^), mean (SD)	23.75 (3.77)	24.14 (3.89)	23.83 (3.87)	23.91 (3.84)
Neutralizing antibody Seropositive^a^, *n* (%)				
HPV 6	101 (38.85)	110 (42.31)	107 (41.15)	318 (40.77)
HPV 11	24 (9.23)	33 (12.69)	34 (13.08)	91 (11.67)
HPV 16	60 (23.08)	56 (21.54)	66 (25.38)	182 (23.33)
HPV 18	42 (16.15)	53 (20.38)	50 (19.23)	145 (18.59)
HPV 31	29 (11.15)	25 (9.62)	26 (10.00)	80 (10.26)
HPV 33	115 (44.23)	118 (45.38)	117 (45.00)	350 (44.87)
HPV 45	54 (20.77)	75 (28.85)	72 (27.69)	201 (25.77)
HPV 52	103 (39.62)	103 (39.62)	110 (42.31)	316 (40.51)
HPV 58	94 (36.15)	93 (35.77)	87 (33.46)	274 (35.13)
IgG antibody seropositive^b^, *n* (%)				
HPV 6	5 (1.92)	7 (2.69)	9 (3.46)	21 (2.69)
HPV 11	4 (1.54)	4 (1.54)	8 (3.08)	16 (2.05)
HPV 16	5 (1.92)	3 (1.15)	9 (3.46)	17 (2.18)
HPV 18	3 (1.15)	4 (1.54)	7 (2.69)	14 (1.79)
HPV 31	2 (0.77)	3 (1.15)	6 (2.31)	11 (1.41)
HPV 33	3 (1.15)	6 (2.31)	7 (2.69)	16 (2.05)
HPV 45	3 (1.15)	1 (0.38)	5 (1.92)	9 (1.15)
HPV 52	2 (0.77)	1 (0.38)	8 (3.08)	11 (1.41)
HPV 58	4 (1.54)	3 (1.15)	10 (3.85)	17 (2.18)

Data are mean (SD) or *n* (%).

*N*, the number of participants in each vaccine group; SD, standard deviation; BMI, body mass index.

a Baseline neutralizing antibody titer ≥ the cutoff value (40).

b Baseline IgG antibody titer ≥ the cutoff value (200).

### 3.2 Safety and tolerability

**Figure 2:**
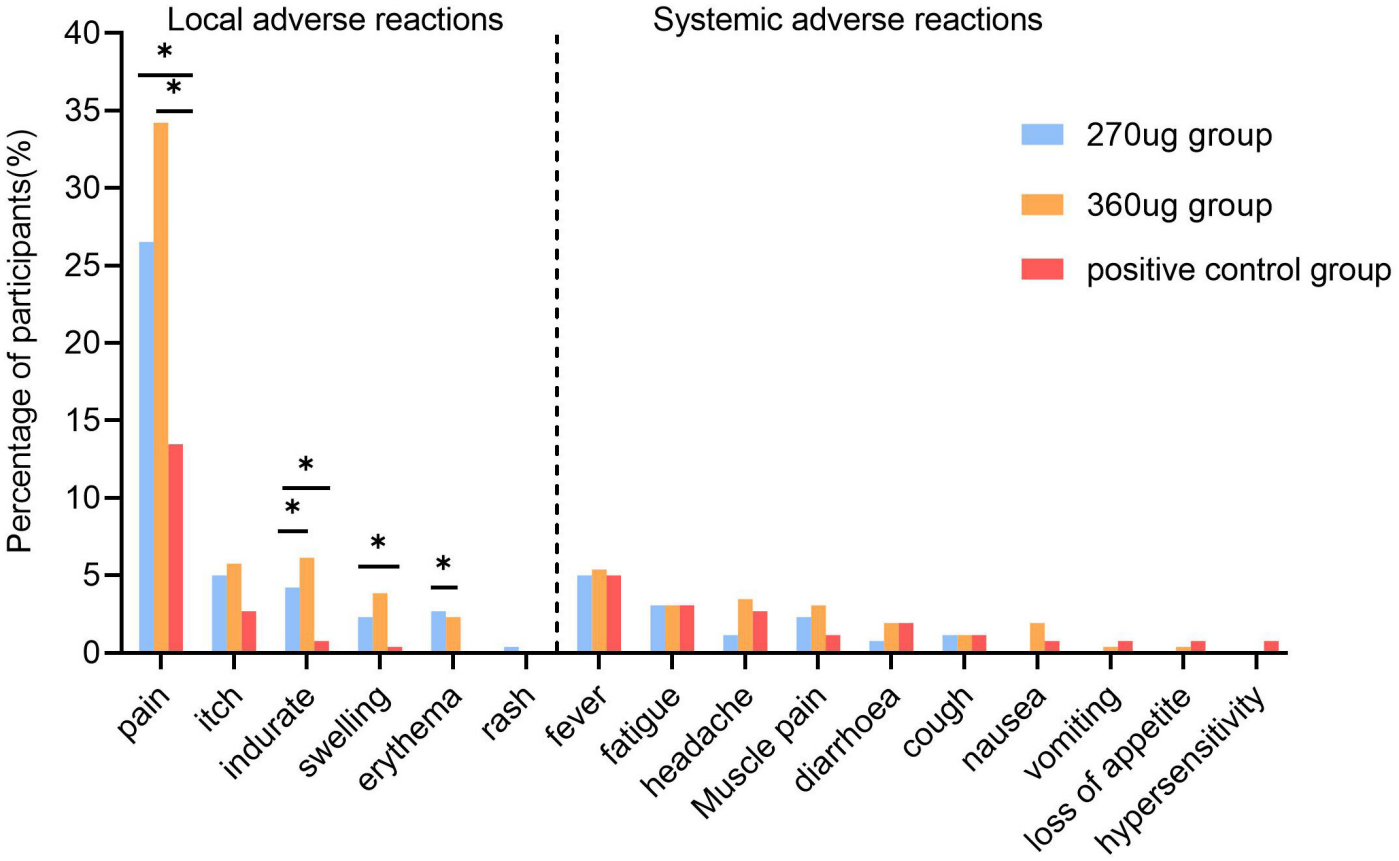
Incidence of solicited adverse reactions within 7 days post-vaccination. The multiple comparisons were adjusted using the Bonferroni method. *a significant difference was found on the basis of adjusted *α* = 0.017.

A total of 269 (34.49%) participants reported at least one AR within 7 days after vaccination. Both the 270 ug group and the 360 ug group showed higher incidences than did the positive control group, with 101 (38.85%), 109 (41.92%), and 59 (22.69%), respectively (*P* < 0.001). The majority of the adverse reactions were mild to moderate. Only one participant in the 270 ug group experienced three grade 3 ARs, which were redness, rash, and swelling at the injection site. ARs occurring within 7 days in each group were mainly solicited ARs. The incidence of solicited ARs was 36.54% in the 270 μg group, 40.77% in the 360 μg group, and 20.77% in the positive control group. Both the 270 ug and the 360 ug groups showed higher incidences than did the positive control group (*P* < 0.001), but no significant difference was found between the 270 ug and 360 ug groups. The most common solicited local AR in the three groups was injection site pain, with 26.4%, 34.23%, and 13.46%, respectively (*P* < 0.001), and the most frequently reported systemic AR was fever, which was reported by 5.00%, 5.38%, and 5.00%, with no statistically significant difference between groups (**Figure 2**). The incidence of unsolicited ARs was comparable among groups, with 2.31%, 3.85%, and 1.92%, respectively (**Supplementary Table S1**).

The overall incidence of AEs within 30 days was 46.54%. The incidence rates in the 270 ug group, the 360 ug group, and the positive control group were 51.54%, 51.92%, and 36.15%, respectively. Both the 270 ug group and the 360 ug group showed higher incidences than did the positive control group (*P* < 0.001). Similarly, the incidences of solicited AEs and solicited local AEs in the 270 ug and 360 ug groups were higher than those in the positive control group. The incidences of unsolicited AEs remained comparable across the three groups, with rates of 24.62%, 23.85%, and 20.00%, respectively. The pattern of AEs within 30 days was similar to ARs that were observed within 7 days, with pain being the most common solicited local AE and fever the most common solicited systemic AE, while the incidence rates of other AEs remained low. There were no deaths reported in the study (**Supplementary Tables S2, S3**). During the study period, a total of nine participants (1.15%) reported 12 SAEs, six participants (2.31%) in the 270 ug group, one (0.38%) in the 360 ug group, and two (0.77%) in the positive control group, with no statistically significant difference (*P* = 0.162). None of such were considered by the investigators to be related to the vaccine (**Supplementary Table S4**).

### 3.3 Immunogenicity

**Figure 3:**
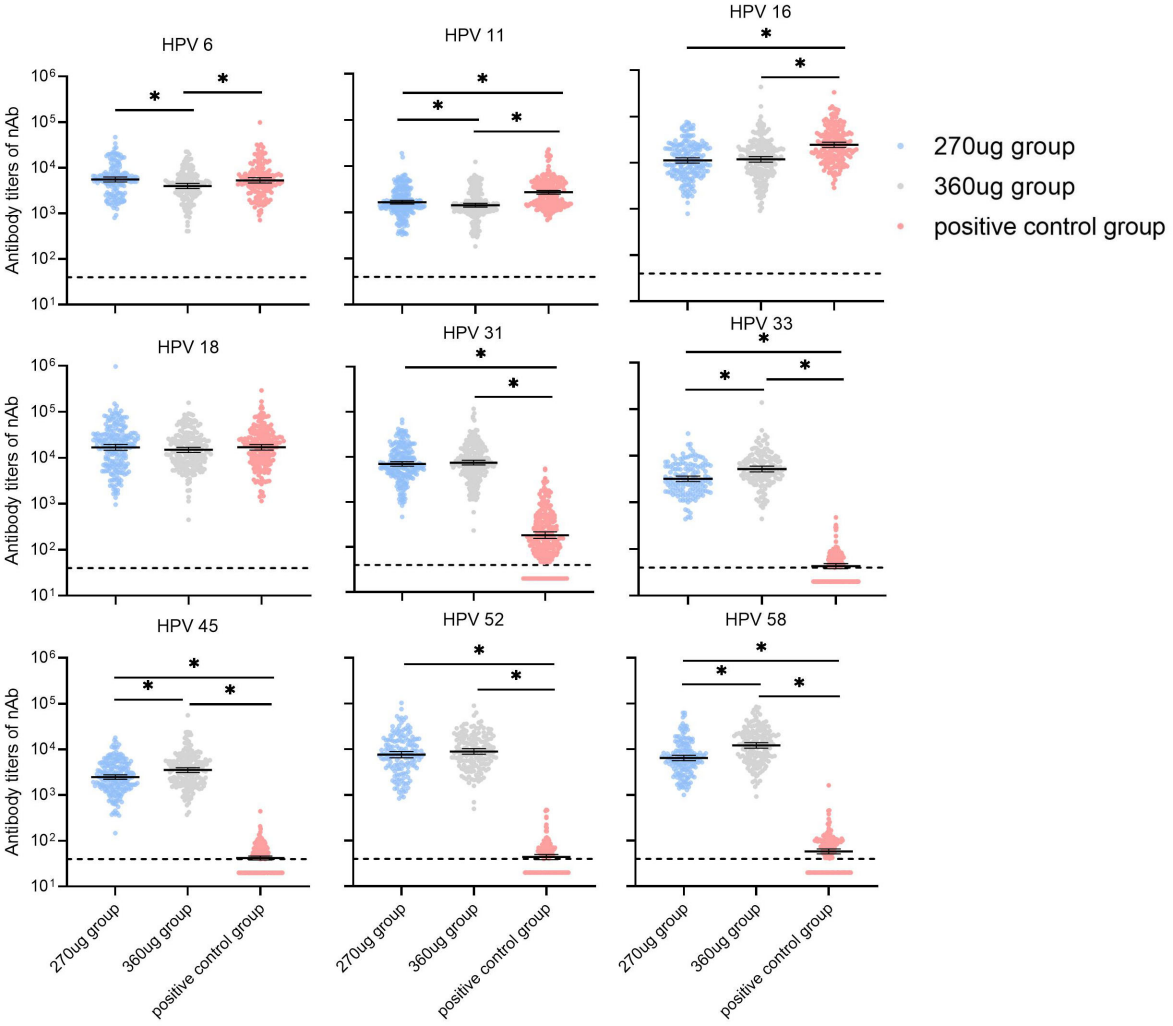
GMTs of neutralizing antibody at month 7 in participants who were baseline seronegative for the corresponding HPV types (PPS). PPS, the per-protocol population includes all participants who received all three vaccinations and donated serum samples at day 0 and month 7 within predefined times windows, with no violation of the protocol. nAb, neutralizing antibody. The dotted lines indicate the cutoff values of the neutralizing antibody (40). The black lines indicate the GMT and 95% CI. Antibody titers below the cutoff value were set as half of the cutoff for GMT calculation. *a significant difference was found on the basis of adjusted *α* = 0.017.

**Figure 4:**
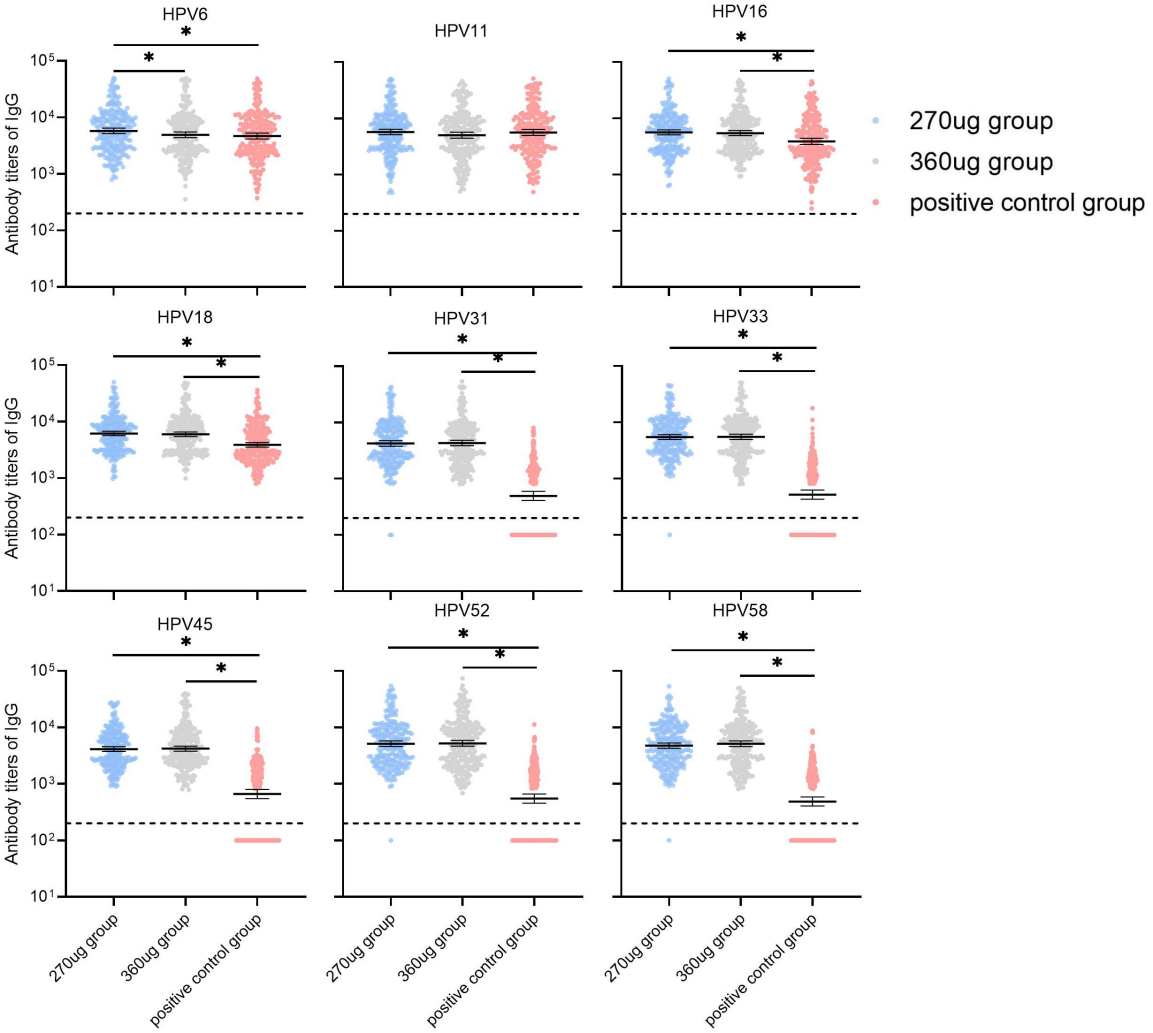
GMTs of IgG antibody at month 7 in participants who were baseline seronegative for the corresponding HPV types (PPS). PPS, the per-protocol population includes all participants who received all three vaccinations and donated serum samples at day 0 and month 7 within predefined times windows, with no violation of the protocol. nAb, neutralizing antibody. The dotted lines indicate the cutoff values of IgG antibody (200). The black lines indicate the GMT and 95% CI. Antibody titers below the cutoff value were set as half of cutoff for GMT calculation. *a significant difference was found on the basis of adjusted α = 0.017.

For participants in the PPS who were nAbs baseline seronegative for relative HPV types, the GMTs of nAbs for HPV types 11 and 16 in the 270 μg group were significantly lower than in the positive control group (*P* < 0.017) and HPV 6, HPV 11, and HPV 16 in the 360 ug group were lower than those in the positive control group (*P* < 0.017). Additionally, it was observed that the nAb GMTs against types 6 and 11 in the 270 ug group were higher than those in the 360 ug group (**Figure 3**). For HPV types 31, 33, 45, 52, and 58, the nAb GMTs in both the 270 μg and 360 μg groups were higher than those in the positive control group. Meanwhile, types 33, 45, and 58 in the 270 ug group were lower than those in the 360 ug group (*P* < 0.017). The immune responses in terms of IgG antibody are shown in **Figure 4**.

Among shared HPV types (HPV 6, HPV 11, HPV 16, and HPV 18), the nAbs GMT ratios ranged from 0.46 to 1.04 in the 270 ug group and 0.48 to 0.88 in the 360 ug group. In the 270 μg group, HPV 6 exhibited the highest GMT ratio at 1.04 (0.87–1.25), while HPV 18 was the highest in the 360 μg group at 0.88 (0.73–1.06). HPV 16 showed the lowest GMT ratios in both dose groups: 0.46 (0.38–0.55) in the 270 μg group and 0.48 (0.40–0.58) in the 360 μg group. Compared to the positive control group, HPV 6 and HPV 18 achieved non-inferiority criteria for GMT in both dose groups (**Table 2**). All participants in the PPS seroconverted for nine HPV types at month 7 after receiving three doses of the candidate vaccine. HPV 6 had the lowest limit of the two-sided 95% CI for the SCR difference at -2.55%. The lower bounds of the two-sided 95% CIs of the differences were all more than the predefined value (–5%) for shared types, and thus the non-inferiority criteria for SCRs were met when compared with the positive control (**Table 3**). Besides that, the GMI of HPV 16 in the 270 μg group was 559.36 (492.43–635.40), HPV 11 and 16 in the 360 μg group were 71.26 (64.78–78.40), and 590.15 (515.14–676.08), respectively (**Supplementary Table S5**). Superiority analyses were performed for HPV types 31, 33, 45, 52, and 58. The results showed that both the 270 μg and 360 μg groups met the superiority criteria, with the lower bounds of the 95% CIs all greater than 1. The largest GMT ratio was 209.47 (173.52–252.87) for type 58 in the 360 ug group and the smallest was 38.15 (31.09–46.83) for type 31 in the 270 ug group (**Table 2**). Besides that, the non-inferiority analysis stratified by age group showed that the GMT ratio for HPV 11 in the 270 μg group was 0.60 (0.50–0.72) in the 20–30 years group, failing to meet the non-inferiority criterion, while the remaining results in this age stratum were consistent with the overall findings. In the 31–45 years group, all results aligned with the overall conclusions (**Supplementary Table S6**). The results of the superiority analysis by age stratum were consistent with the overall results, demonstrating that the candidate vaccine was superior to the control vaccine in all age groups (**Supplementary Table S7**).

**Table 2:** Non-inferiority and superiority analysis of neutralizing antibody GMT levels at month 7 in participants who were baseline seronegative for the corresponding HPV types (PPS).

HPV type	270 ug (*N* = 254)		360 ug (*N* = 256)		Positive control (*N* = 251)		GMT ratio (95% CI) [270 ug/positive control]	GMT ratio (95% CI) [360 ug/positive control]
	n	GMT (95% CI)	n	GMT (95% CI)	n	GMT (95% CI)		
HPV 6	156	5,540.59 (4,886.31, 6,282.48)	147	3,977.96 (3,492.88, 4,530.41)	149	5,310.99 (4,646.59, 6,070.39)	1.04 (0.87, 1.25)	0.75 (0.62, 0.90)
HPV 11	230	1,655.82 (1,510.69, 1,814.91)	223	1,425.30 (1,295.60, 1,567.98)	217	2,717.16 (2,469.45, 2,989.72)	0.61 (0.53, 0.70)	0.53 (0.46, 0.60)
HPV 16	197	11,187.29 (9,848.56, 12,707.99)	201	11,803.01 (10,302.81, 13,521.65)	186	24,370.41 (21,477.89, 27,652.48)	0.46 (0.38, 0.55)	0.48 (0.40, 0.58)
HPV 18	214	16 841.82 (14,524.93, 19,528.27)	204	14,900.76 (13,115.67, 16,928.81)	201	16,915.79 (14,729.42, 19,426.68)	1.00 (0.81, 1.22)	0.88 (0.73, 1.06)
HPV 31	225	6,999.33 (6224.37,7870.77)	231	7,465.20 (6,611.99, 8,428.50)	226	183.45 (155.01, 217.12)	38.15 (31.09, 46.83)	40.69 (33.08, 50.06)
HPV 33	143	3,208.33 (2,813.97, 3,657.96)	140	5,190.10 (4,506.10, 5,977.93)	138	42.86 (37.95, 48.40)	74.86 (62.63, 89.49)	121.10 (100.56,145.85)
HPV 45	203	2,481.32 (2,215.89, 2778.55)	182	3,527.70 (3,118.83, 3,990.18)	181	42.20 (38.26, 46.55)	58.80 (50.65, 68.26)	83.59 (71.45,97,79)
HPV 52	155	7,634.70 (6,528.38, 8,928.52)	155	8,909.80 (7,741.32, 10,254.65)	144	43.88 (38.56, 49.95)	173.97 (142.10, 212.99)	203.03 (167.70, 245.80)
HPV 58	164	6,453.63 (5,656.31, 7,363.34)	165	12,150.21 (10,571.65, 13,964.49)	166	58.00 (51.04, 65.92)	111.26 (92.66, 133.61)	209.47 (173.52, 252.87)

The 9vHPV vaccine was considered non-inferior to positive control vaccine if the lower limit of the 95% CI for the GMT ratio is greater than 0.5 between shared types (HPV 6, 11, 16, 18). For HPV 31, 33, 45, 52, 58, superiority is demonstrated if the lower limit of the 95% CI for the GMT ratio of antibody titers is greater than 1.

GMT, geometric mean titer; PPS, the per-protocol population includes all participants who received all three vaccinations and donated serum samples at day 0 and month 7 within predefined times windows, with no violation of the protocol; *N*, the number of participants in each group; *n*, the number of participants in each group who were baseline seronegative of neutralizing antibody for the corresponding HPV types.

**Table 3:** Seroconversion rates of neutralizing antibody at month 7 in participants who were baseline seronegative for the corresponding HPV types (PPS).

HPV type	270 ug		360 ug		Positive control		Difference in SCR (95% CI) [270 ug—positive control]	Difference in SCR (95% CI) [360 ug—positive control]
	n/N	SCR, % (95% CI)	n/N	SCR, % (95% CI)	n/N	SCR, % (95% CI)		
HPV 6	156/156	100.00 (97.60, 100.00)	147/147	100.00 (97.45, 100.00)	149/149	100.00 (97.49, 100.00)	0 (-2.40, 2.51)	0 (-2.55, 2.51)
HPV 11	230/230	100.00 (98.36, 100.00)	223/223	100.00 (98.31, 100.00)	217/217	100.00 (98.26, 100.00)	0 (-1.64, 1.74)	0 (-1.69, 1.74)
HPV 16	197/197	100.00 (98.09, 100.00)	201/201	100.00 (98.12, 100.00)	186/186	100.00 (97.98, 100.00)	0 (-1.91, 2.02)	0 (-1.88, 2.02)
HPV 18	214/214	100.00 (98.24, 100.00)	204/204	100.00 (98.15, 100.00)	201/201	100.00 (98.12, 100.00)	0 (-1.76, 1.88)	0 (-1.85, 1.88)
HPV 31	225/225	100.00 (98.32, 100.00)	231/231	100.00 (98.36, 100.00)	202/226	89.38 (84.69, 92.76)	10.62 (6.58, 14.66)	10.62 (6.72, 14.52)
HPV 33	143/143	100.00 (98.38, 100.00)	140/140	100.00 (97.33, 100.00)	84/138	60.87 (52.54, 68.61)	39.13 (31.50, 46.33)	39.13 (31.30, 46.56)
HPV 45	203/203	100.00 (98.14, 100.00)	182/182	100.00 (97.93, 100.00)	111/181	61.33 (54.07, 68.12)	38.67 (31.24, 45.57)	38.67 (31.04, 45.86)
HPV 52	155/155	100.00 (97.58, 100.00)	155/155	100.00 (97.58, 100.00)	84/144	58.33 (50.17, 66.07)	41.67 (32.90, 50.04)	41.67 (32.90, 50.04)
HPV 58	164/164	100.00 (97.71, 100.00)	155/155	100.00 (97.72, 100.00)	116/166	69.88 (62.52, 76.34)	30.12 (21.37, 38.45)	30.12 (21.05, 38.83)

Two-sided 95% CIs of the SCR differences were computed using the Miettine–Nurminen method, and the Newcombe method was used when both groups had 100% of SCRs.

PPS, the per-protocol population includes all participants who received all three vaccinations and donated serum samples at day 0 and month 7 within predefined times windows, with no violation of the protocol; SCR, seroconversion rate; *N*, the number of participants in PPS who were baseline seronegative for the corresponding HPV type; *n*, the number of participants who seroconverted (having four times or higher increase of antibody titers) for corresponding HPV type at month 7 in PPS.

For all participants in the PPS, the GMTs of nAbs for all nine HPV types in both 270 ug and 360 ug groups showed an obvious rise at month 7, while there was only a sharp surge for HPV 6, HPV 11, HPV 16, and HPV 18 in the positive control group (**Supplementary Figure S2**). Similar trends were also observed in terms of IgG antibody (**Supplementary Figure S3**). Almost all participants seroconverted for nAbs and IgG antibodies against all nine HPV types at month 7 in the test groups. As for nAbs, one failed to seroconvert for HPV 18, two for HPV 31, one for HPV 33, two for HPV 45, one for HPV 52, and one for HPV 58 in the 270 ug group. One failed for HPV 16, two for HPV 45, and one for HPV 52 in the 360 ug group. All participants seroconverted for IgG antibody against all nine HPV types in the 360 ug group, while two failed to seroconvert for HPV 31, one for HPV 33, one for HPV 52, and one for HPV 58 in the 270 ug group. In the positive control group, all participants achieved seroconversion of both nAbs and IgG antibodies against HPV 6, HPV 11, HPV 16, and HPV 18. Notably, a relatively high SCR of non-vaccine types can be observed in the positive control group. However, the GMIs are lower compared with the candidate 9vHPV vaccine (**Supplementary Table S8**).

Finally, we conducted an additional sensitivity analysis. The results were consistent with those observed in the participants who were nAbs seronegative for the corresponding HPV type at baseline (**Supplementary Table S9**). The exploratory analysis results indicated that the SCRs for GST and 3C protease antibodies at month 3 and month 7 were 0 in the three vaccine groups.

## 4 Discussion

This phase 2 clinical trial indicated that the candidate *E. coli*-produced 9vHPV vaccine was well tolerated and immunogenic in healthy women aged 20–45 years old. After a three-dose regimen, three groups elicited robust antibody responses and showed good safety. For shared HPV types 6, 11, 16, and 18, both dose groups of candidate 9vHPV vaccine demonstrated immunological non-inferiority for GMT compared with the positive control vaccine, except HPV 16 in the 270 μg group and HPV 11 and HPV 16 in the 360 μg group. However, HPV 16 in the 270 μg group as well as HPV 11 and HPV 16 in the 360 μg group all met the non-inferiority criteria for SCR. For the additional HPV types 31, 33, 45, 52, and 58, the candidate vaccine achieved superiority criteria for GMT compared with the positive control vaccine.

The 9vHPV vaccine was generally well tolerated, and the characteristics of AEs were similar to those of the control vaccine. The incidence of total ARs within 7 days in the 270 μg, 360 μg, and positive control groups was 38.85%, 41.92%, and 22.69%, respectively, which is similar to the results reported in a previous phase 1 clinical trial of an *E. coli*-based bivalent HPV vaccine (15). The incidence of AEs and ARs in the 9-valent vaccine was higher than that in the qHPV, which is consistent with the characteristics of other multivalent HPV vaccines (16). This may be attributed to the increased antigen content (270 ug or 360 ug for the candidate vaccine and 120 ug for the control vaccine) and the increased number of antigen types. Studies have shown that the higher incidence of ARs of the 9vHPV vaccine compared with the control vaccine may be related to the increase in the antigen content. The higher VLP content, the higher is incidence of ARs after vaccination (16). Although the incidence of ARs in the 360 ug group is slightly higher numerically than that in the 270 ug group, there was no statistically significant difference between the two groups. The symptoms of ARs mainly concentrate at the injection site, and the most common one is pain, which is consistent with the research results of the *Escherichia coli*-based 9vHPV vaccine Cecolin 9 (17, 18). Moreover, the vast majority of adverse events were mild to moderate, and no vaccine-related SAEs occurred. Additionally, the exploratory analyses indicated that the SCRs for GST and 3C protease antibodies at month 3 and month 7 were 0 following vaccination across all study groups, which further demonstrated the safety of the candidate vaccine.

Studies have shown that the HPV vaccine mainly provides protection against HPV infection by generating and enriching nAbs (19). A strong immunogenic response was detected after three doses of 9vHPV vaccine. Nearly all participants in the PPS developed seroconversion of nAbs and IgG antibodies against nine HPV types, which is similar to the findings from the phase 3 study of Gardasil 9 (99.60%–100.00%) (20). The GMIs of nAbs and IgG antibodies reached several tens or even several hundreds of times, which is consistent with the results of the phase I study of this vaccine (21). In this study, we also observed a relatively high SCR in the positive control group for non-vaccine HPV types. Considering the cross-protection among HPV types, such as the pairs of HPV 16 and HPV 31, HPV 18 and HPV 45, and HPV 6 and HPV 11, which share a high degree of structural similarity, they likely possess identical or similar antigenic epitopes. These epitopes can be recognized and neutralized by the same antibody, thus giving rise to a cross-neutralization effect (19).

The GMTs of nAbs against HPV 16 in the 270 ug group and HPV 11 and HPV 16 in the 360 ug group at month 7 did not meet the non-inferiority criteria for GMT. The underlying causes are postulated to be the potential immunological effect reallocation and immunological interference among various HPV types inherent in high-valence HPV vaccines (22, 23). However, all of the subjects experienced seroconversion of nAbs against HPV 16 and HPV 11 and had relatively high antibody levels at month 7. Besides that, both groups met the non-inferiority criteria for SCR. The GMI of nAbs against HPV 16 in the 270 ug group was 559.36 (492.43–635.40) and 71.26 (64.78–78.40) and 590.15 (515.14–676.08) against HPV 11 and HPV 16, respectively, in the 360 ug group. These results indicate that although the GMT non-inferiority criteria were not met for these HPV types, a robust immune response was generated after three doses of 9vHPV vaccine. Additionally, GMTs only reflect antibody levels and cannot fully represent a vaccine’s protective efficacy as vaccines can provide long-term protection through immune memory. A study evaluated the protective efficacy of the bivalent HPV vaccine across different dose regimens. The results showed that the three-dose regimen induced the highest antibody levels, followed by the two-dose regimen, with the single-dose regimen resulting in the lowest levels. Specifically, the antibody GMT in the single-dose group was significantly lower than that in the multi-dose groups (*P* < 0.001). However, the vaccine efficacy in the single-dose group was comparable to that in the multi-dose groups (24). Therefore, further verification will be carried out in the phase 3 clinical trial, which is currently undergoing its 48th month of follow-up. Compared with the 360 ug group, the 270 ug group showed slightly higher nAb levels against HPV 6 and HPV 11, slightly lower levels against HPV 33, HPV 45, and HPV 58, and no statistically significant differences for other types. Considering that HPV types 33, 45, and 58, respectively, account for 4%, 6%, and 2% of cervical cancers, while HPV types 6 and 11 cause 90% of anogenital warts (7), the immunogenic advantage of 360 ug is not obvious. Moreover, the incidence of ARs was similar between the two groups. Therefore, 270 ug is expected to be the optimal dosage for future study.

A key strength of this study is the use of qHPV as positive control. Although a placebo control could theoretically provide stronger statistical power to demonstrate the test vaccine’s immunogenicity, this would be inconsistent with ethical requirements. The primary objective of the phase 2 trial is to evaluate the candidate 9vHPV at two doses and select the optimal dose for progression to the phase 3 efficacy trial. Since Gardasil shares four HPV types with the candidate vaccine, a direct non-inferiority comparison can be conducted, which serves as the most direct basis for dose selection. The additional five types can be evaluated through superiority analysis, thus enabling a combined “non-inferiority–superiority” statistical strategy. Meanwhile, considering vaccine accessibility, cost, and its extensive application basis in the Chinese population, selecting the quadrivalent vaccine as the control was reasonable and ethical. Furthermore, we had used Gardasil 9 as the control in the phase 1 clinical trial, which preliminarily verified the comparability of the candidate vaccine with the world’s first 9vHPV in terms of safety and immunogenicity. However, there are also several limitations in our study. First, the study population was restricted to women aged 20–45 years, potentially limiting the generalizability of findings to other demographic groups that may benefit from HPV immunization. Future research should incorporate younger populations who represent a key target group for prophylactic HPV vaccination. Second, in the analysis of the primary immunogenicity outcomes, a per-protocol analysis was employed, excluding individuals who did not complete the full vaccination course and those with missing serum antibody results, which may lead to a potential selection bias. In addition, compared to other clinical trials, this study showed higher baseline nAbs seropositivity rates across HPV types, which are likely due to different detection methods. Moreover, we also observed that the baseline seropositivity rates of nAbs detected by the PBNA was significantly higher than that of IgG detected by ELISA. This difference is most likely attributed to the differences in detection threshold settings and principles between the two assays. The PBNA is intended to detect functionally active antibodies, and its detection threshold is lower than that for IgG, which may lead to a higher seropositivity rate for low-level immunity induced by previous natural infections. Notably, the baseline difference was balanced across all vaccine groups, and our primary immunogenicity analysis was based on participants who were baseline seronegative for nAbs. In addition, the subgroup analysis of the entire population and the sensitivity analysis based on baseline IgG seronegativity showed immune response trends consistent with the primary analysis, indicating that the differences did not affect the core conclusion regarding the immunogenicity of the candidate vaccine.

In conclusion, the study has demonstrated that the candidate *E. coli*-produced 9vHPV vaccine was well tolerated and immunogenic, which encourages further safety, immunogenicity, and efficacy studies in large populations.

## Data Availability

The raw data supporting the conclusions of this article will be made available by the authors, without undue reservation.
