# Supplementary material for: Immunogenicity and safety of an Escherichia coli-produced 9-valent human papillomavirus vaccine (types 6/11/16/18/31/33/45/52/58) in healthy Chinese women aged 20–45 years: a single-center, randomized, observer-blinded, positive controlled phase 2 clinical trial
Source: Front Immunol. 2025 Nov 14;16:1706662. doi: 10.3389/fimmu.2025.1706662 (PMC12661565; doi:10.3389/fimmu.2025.1706662)
Supplement: Supplementary file 1 [file DataSheet1.docx]

**Supplementary information**


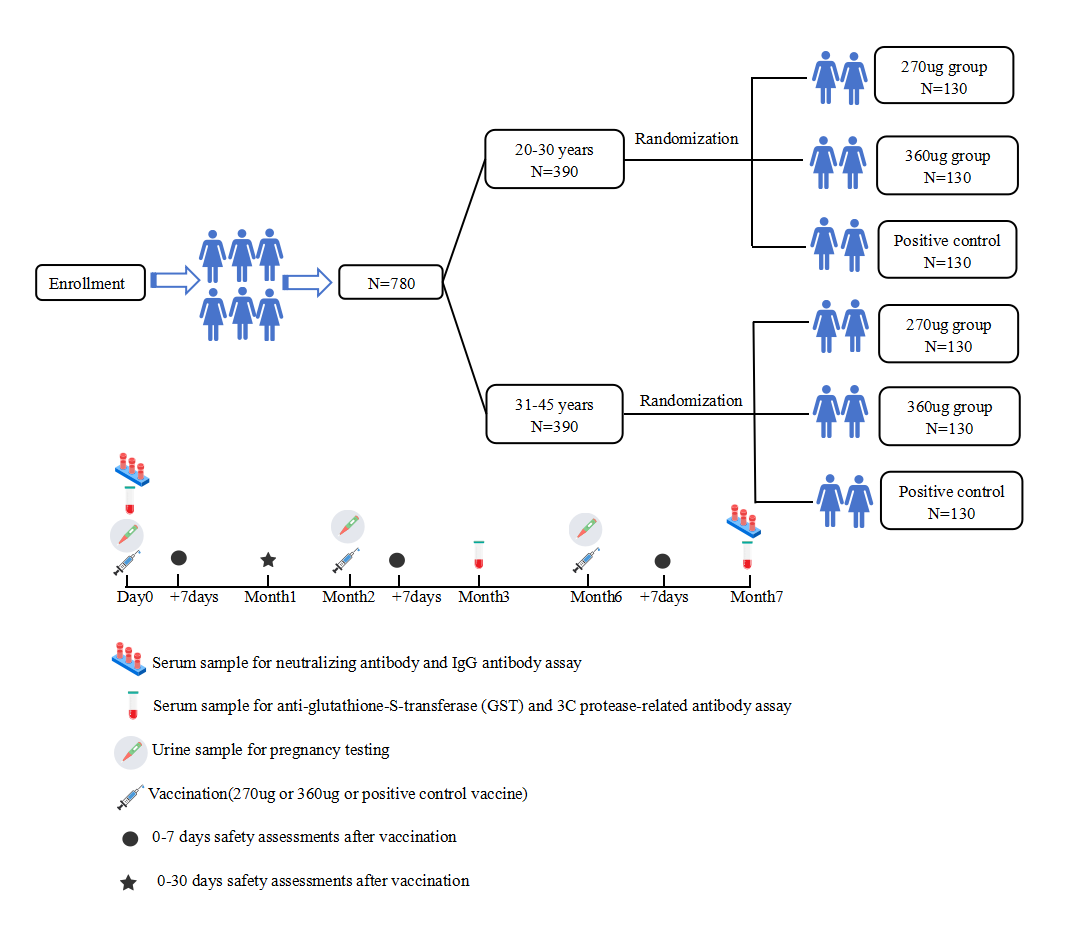


**Figure S1. Overview of study design.** N=the number of participants. qHPV: quadrivalent human papillomavirus vaccine.

**Table S1. Grades of adverse reactions within 7 days post-vaccination.**

|  | 270ug  (N=260) | | 360ug  (N=260) | | positive control  (N=260) | | Total  (N=780) | | ***p* value*** |
| --- | --- | --- | --- | --- | --- | --- | --- | --- | --- |
|  | Participants(%) | Events | Participants(%) | Events | Participants(%) | Events | Participants(%) | Events |  |
| **Any** | 101(38.85) | 185 | 109(41.92) | 251 | 59(22.69) | 112 | 269(34.49) | 548 | <0.001^**^ |
| Grade 1 | 99(38.08) | 176 | 108(41.54) | 238 | 58(22.31) | 106 | 265(33.97) | 520 | <0.001^**^ |
| Grade 2 | 4(1.54) | 6 | 9(3.46) | 13 | 5(1.92) | 6 | 18(2.31) | 25 | 0.303 |
| ≥Grade 3 | 1(0.38) | 3 | 0 | 0 | 0 | 0 | 1(0.13) | 3 | >0.999 |
| **Solicited** | 95(36.54) | 179 | 106(40.77) | 237 | 54(20.77) | 107 | 255(32.69) | 523 | <0.001^**^ |
| Grade 1 | 94(36.150) | 171 | 105(40.38) | 225 | 53(20.38) | 101 | 252(32.31) | 497 | <0.001^**^ |
| Grade 2 | 3(1.15) | 5 | 8(3.08) | 12 | 5(1.92) | 6 | 16(2.05) | 23 | 0.297 |
| ≥Grade 3 | 1(0.38) | 3 | 0 | 0 | 0 | 0 | 1(0.13) | 3 | >0.999 |
| **Local site** | 78(30.00) | 140 | 96(36.92) | 178 | 36(13.85) | 58 | 210(26.92) | 376 | <0.001^**^ |
| Grade 1 | 77(29.62) | 132 | 95(36.54) | 167 | 35(13.46) | 56 | 207(26.54) | 355 | <0.001^**^ |
| Grade 2 | 3(1.15) | 5 | 7(2.69) | 11 | 2(0.77) | 2 | 12(1.54) | 18 | 0.262 |
| ≥Grade 3 | 1(0.38) | 3 | 0 | 0 | 0 | 0 | 1(0.13) | 3 | >0.999 |
| **Systemic** | 28(10.77) | 39 | 35(13.46) | 59 | 32(12.31) | 49 | 95(12.18) | 147 | 0.642 |
| Grade 1 | 28(10.77) | 39 | 34(13.08) | 58 | 31(11.92) | 45 | 93(11.92) | 142 | 0.719 |
| Grade 2 | 0 | 0 | 1(0.38) | 1 | 3(1.15) | 4 | 4(0.51) | 5 | 0.332 |
| ≥Grade 3 | 0 | 0 | 0 | 0 | 0 | 0 | 0 | 0 | - |
| **Unsolicited** | 6(2.31) | 6 | 10(3.85) | 14 | 5(1.92) | 5 | 21(2.69) | 25 | 0.358 |
| Grade 1 | 5(1.92) | 5 | 9(3.46) | 13 | 5(1.92) | 5 | 19(2.44) | 23 | 0.422 |
| Grade 2 | 1(0.38) | 1 | 1(0.38) | 1 | 0 | 0 | 2(0.26) | 2 | >0.999 |
| ≥Grade 3 | 0 | 0 | 0 | 0 | 0 | 0 | 0 | 0 | - |

N=the number of participant in each group. Grade 1 was a mild reaction, grade 2 was a moderate reaction, and grade 3 was a severe reaction. *p values were generated from the comparison across the three groups, which were calculated using Pearson’s chi-squared test or Fisher’s exact test. When significant difference across vaccine groups was found,we further applied pairwise comparisons on the basis of adjusted ɑ=0.017. **Both the 270ug and 360ug groups showed higher incidences than did the positive control group,but no significant difference was noted between the 270ug and 360ug groups.

**Table S2. Summary of adverse events within 30 days post-vaccination.**

|  | 270ug  (N=260) | | 360ug  (N=260) | | positive control  (N=260) | | Total  (N=780) | | ***p* value*** |
| --- | --- | --- | --- | --- | --- | --- | --- | --- | --- |
|  | Participants(%) | Events | Participants(%) | Events | Participants(%) | Events | Participants(%) | Events |  |
| Total adverse events | 134(51.54) | 269 | 135(51.92) | 324 | 94(36.15) | 180 | 363(46.54) | 773 | <0.001^**^ |
| Total adverse events≥grade 3 | 4(1.54) | 7 | 0 | 0 | 1(0.38) | 1 | 5(0.64) | 8 | 0.134 |
| Total adverse reactions^a^ | 101(38.85) | 190 | 109(41.92) | 252 | 59(22.69) | 113 | 269(34.49) | 555 | <0.001^**^ |
| Total adverse reactions^a^≥grade 3 | 1(0.38) | 3 | 0 | 0 | 0 | 0 | 1(0.13) | 3 | >0.999 |
| Solicited adverse events | 95(36.54) | 180 | 107(41.15) | 239 | 54(20.77) | 107 | 256(32.82) | 526 | <0.001^**^ |
| Local adverse events | 78(30.00) | 141 | 96(36.92) | 178 | 36(13.85) | 58 | 210(26.92) | 377 | <0.001^**^ |
| Systemic adverse events | 28(10.77) | 39 | 36(13.85) | 61 | 32(12.31) | 49 | 96(12.31) | 149 | 0.565 |
| Unsolicited adverse events | 64(24.62) | 89 | 62(23.85) | 85 | 52(20.00) | 73 | 178(22.82) | 247 | 0.406 |
| Withdrawal due to adverse events | 0 | 0 | 0 | 0 | 1(0.38) | 1 | 1(0.13) | 1 | >0.999 |
| Withdrawal due to adverse reactions | 0 | 0 | 0 | 0 | 0 | 0 | 0 | 0 | - |
| Serious adverse events | 6(2.31) | 8 | 1(0.38) | 2 | 2(0.77) | 2 | 9(1.15) | 12 | 0.162 |
| Serious adverse reactions | 0 | 0 | 0 | 0 | 0 | 0 | 0 | 0 | - |

N=the number of participants in each vaccine group. qHPV: quadrivalent human papillomavirus vaccine. ^a^ Definitely related, likely related, possibly related defined as adverse reactions. *p values were generated from the comparison across the three groups, which were calculated using Pearson’s chi-squared test or Fisher’s exact test. When significant difference across vaccine groups was found, we further applied pairwise comparisons on the basis of adjusted ɑ=0.017. **Both the 270ug and 360ug groups showed higher incidences than did the qHPV vaccine group, but no significant difference was noted between the 270ug and 360ug groups.

**Table S3. Grades of adverse events within 30 days post-vaccination.**

|  | 270ug (N=260) | | 360ug (N=260) | | positive control  (N=260) | | Total  (N=780) | | ***p* value*** |
| --- | --- | --- | --- | --- | --- | --- | --- | --- | --- |
|  | Participants  (%) | Events | Participants  (%) | Events | Participants  (%) | Events | Participants  (%) | Events |  |
| **Any** | 134(51.54) | 269 | 135(51.92) | 324 | 94(36.15) | 180 | 363(46.54) | 773 | <0.001^**^ |
| Grade 1 | 118(45.38) | 225 | 125(48.08) | 286 | 74(28.46) | 140 | 317(40.64) | 651 | <0.001^**^ |
| Grade 2 | 29(11.15) | 37 | 30(11.54) | 38 | 31(11.92) | 38 | 90(11.54) | 113 | 0.963 |
| ≥Grade 3 | 4(1.54) | 7 | 0 | 0 | 1(0.38) | 1 | 5(0.64) | 8 | 0.134 |
| **Solicited** | 95(36.54) | 180 | 107(41.15) | 239 | 54(20.77) | 107 | 256(32.82) | 526 | <0.001^**^ |
| Grade 1 | 94(36.15) | 171 | 105(40.38) | 226 | 53(20.38) | 101 | 252(32.31) | 498 | <0.001 |
| Grade 2 | 4(1.54) | 6 | 9(3.46) | 13 | 5(1.92) | 6 | 18(2.31) | 25 | 0.303 |
| ≥Grade 3 | 1(0.38) | 3 | 0 | 0 | 0 | 0 | 1(0.13) | 3 | >0.999 |
| **Local site** | 78(30.00) | 141 | 96(36.92) | 178 | 36(13.85) | 58 | 210(26.92) | 377 | <0.001^**^ |
| Grade 1 | 77(29.62) | 132 | 95(36.54) | 167 | 35(13.46) | 56 | 207(26.54) | 355 | <0.001^**^ |
| Grade 2 | 4(1.54) | 6 | 7(2.69) | 11 | 2(0.77) | 2 | 13(1.67) | 19 | 0.263 |
| ≥Grade 3 | 1(0.38) | 3 | 0 | 0 | 0 | 0 | 1(0.13) | 3 | >0.999 |
| **Pain** | 69(26.54) | 99 | 89(34.23) | 128 | 35(13.46) | 48 | 193(24.74) | 275 | <0.001^**^ |
| Grade 1 | 69(26.54) | 99 | 87(33.46) | 126 | 34(13.08) | 47 | 190(24.36) | 272 | <0.001^**^ |
| Grade 2 | 0 | 0 | 2(0.77) | 2 | 1(0.38) | 1 | 3(0.38) | 3 | 0.777 |
| ≥Grade 3 | 0 | 0 | 0 | 0 | 0 | 0 | 0 | 0 | - |
| **Itch** | 13(5.00) | 16 | 15(5.77) | 17 | 7(2.69) | 7 | 35(4.49) | 40 | 0.211 |
| Grade 1 | 13(5.00) | 16 | 15(5.77) | 17 | 7(2.69) | 7 | 35(4.49) | 40 | 0.211 |
| Grade 2 | 0 | 0 | 0 | 0 | 0 | 0 | 0 | 0 | - |
| ≥Grade 3 | 0 | 0 | 0 | 0 | 0 | 0 | 0 | 0 | - |
| **Indurate** | 12(4.62) | 12 | 16(6.15) | 17 | 2(0.77) | 2 | 30(3.85) | 31 | <0.001^**^ |
| Grade 1 | 8(3.08) | 8 | 13(5.00) | 14 | 2(0.77) | 2 | 23(2.95) | 24 | 0.017^**^ |
| Grade 2 | 4(1.54) | 4 | 3(1.15) | 3 | 0 | 0 | 7(0.90) | 7 | 0.172 |
| ≥Grade 3 | 0 | 0 | 0 | 0 | 0 | 0 | 0 | 0 | - |
| **Swelling** | 6(2.31) | 6 | 10(3.85) | 10 | 1(0.38) | 1 | 17(2.18) | 17 | 0.026 |
| Grade 1 | 4(1.54) | 4 | 5(1.92) | 5 | 0 | 0 | 9(1.15) | 9 | 0.087 |
| Grade 2 | 1(0.38) | 1 | 5(1.92) | 5 | 1(0.38) | 1 | 7(0.90) | 7 | 0.229 |
| ≥Grade 3 | 1(0.38) | 1 | 0 | 0 | 0 | 0 | 1(0.13) | 1 | >0.999 |
| **Erythema** | 7(2.69) | 7 | 6(2.31) | 6 | 0 | 0 | 13(1.67) | 13 | 0.018 |
| Grade 1 | 5(1.92) | 5 | 5(1.92) | 5 | 0 | 0 | 10(1.28) | 10 | 0.055 |
| Grade 2 | 1(0.38) | 1 | 1(0.38) | 1 | 0 | 0 | 2(0.26) | 2 | >0.999 |
| ≥Grade 3 | 1(0.38) | 1 | 0 | 0 | 0 | 0 | 1(0.13) | 1 | >0.999 |
| **Rash** | 1(0.38) | 1 | 0 | 0 | 0 | 0 | 1(0.13) | 1 | >0.999 |
| Grade 1 | 0 | 0 | 0 | 0 | 0 | 0 | 0 | 0 | - |
| Grade 2 | 0 | 0 | 0 | 0 | 0 | 0 | 0 | 0 | - |
| ≥Grade 3 | 1(0.38) | 1 | 0 | 0 | 0 | 0 | 1(0.13) | 1 | >0.999 |
| **Systemic** | 28(10.77) | 39 | 36(13.85) | 61 | 32(12.31) | 49 | 96(12.31) | 149 | 0.565 |
| Grade 1 | 28(10.77) | 39 | 34(13.08) | 59 | 31(11.92) | 45 | 93(11.92) | 143 | 0.719 |
| Grade 2 | 0 | 0 | 2(0.77) | 2 | 3(1.15) | 4 | 5(0.64) | 6 | 0.380 |
| ≥Grade 3 | 0 | 0 | 0 | 0 | 0 | 0 | 0 | 0 | - |
| **Fever** | 13(5.00) | 15 | 14(5.38) | 18 | 13(5.00) | 15 | 40(5.13) | 48 | 0.974 |
| Grade 1 | 13(5.00) | 15 | 14(5.38) | 18 | 13(5.00) | 15 | 40(5.13) | 48 | 0.974 |
| Grade 2 | 0 | 0 | 0 | 0 | 0 | 0 | 0 | 0 | - |
| ≥Grade 3 | 0 | 0 | 0 | 0 | 0 | 0 | 0 | 0 | - |
| **Fatigue** | 8(3.08) | 9 | 8(3.08) | 9 | 8(3.08) | 8 | 24(3.08) | 26 | >0.999 |
| Grade 1 | 8(3.08) | 9 | 8(3.08) | 9 | 8(3.08) | 8 | 24(3.08) | 26 | >0.999 |
| Grade 2 | 0 | 0 | 0 | 0 | 0 | 0 | 0 | 0 | - |
| ≥Grade 3 | 0 | 0 | 0 | 0 | 0 | 0 | 0 | 0 | - |
| **Headache** | 3(1.15) | 3 | 10(3.85) | 10 | 7(2.69) | 7 | 20(2.56) | 20 | 0.150 |
| Grade 1 | 3(1.15) | 3 | 9(3.46) | 9 | 7(2.69) | 7 | 19(2.44) | 19 | 0.221 |
| Grade 2 | 0 | 0 | 1(0.38) | 1 | 0 | 0 | 1(0.13) | 1 | >0.999 |
| ≥Grade 3 | 0 | 0 | 0 | 0 | 0 | 0 | 0 | 0 | - |
| **Muscle pain** | 6(2.31) | 7 | 8(3.08) | 8 | 3(1.15) | 3 | 17(2.18) | 18 | 0.319 |
| Grade 1 | 6(2.31) | 7 | 7(2.69) | 7 | 2(0.77) | 2 | 15(1.92) | 16 | 0.240 |
| Grade 2 | 0 | 0 | 1(0.38) | 1 | 1(0.38) | 1 | 2(0.26) | 2 | >0.999 |
| ≥Grade 3 | 0 | 0 | 0 | 0 | 0 | 0 | 0 | 0 | - |
| **Diarrhoea** | 2(0.77) | 2 | 5(1.92) | 5 | 5(1.92) | 5 | 12(1.54) | 12 | 0.512 |
| Grade 1 | 2(0.77) | 2 | 5(1.92) | 5 | 5(1.92) | 5 | 12(1.54) | 12 | 0.512 |
| Grade 2 | 0 | 0 | 0 | 0 | 0 | 0 | 0 | 0 | - |
| ≥Grade 3 | 0 | 0 | 0 | 0 | 0 | 0 | 0 | 0 | - |
| **Cough** | 3(1.15) | 3 | 3(1.15) | 4 | 3(1.15) | 3 | 9(1.15) | 10 | >0.999 |
| Grade 1 | 3(1.15) | 3 | 3(1.15) | 4 | 3(1.15) | 3 | 9(1.15) | 10 | >0.999 |
| Grade 2 | 0 | 0 | 0 | 0 | 0 | 0 | 0 | 0 | - |
| ≥Grade 3 | 0 | 0 | 0 | 0 | 0 | 0 | 0 | 0 | - |
| **Naused** | 0 | 0 | 5(1.92) | 5 | 2(0.77) | 2 | 7(0.90) | 7 | 0.077 |
| Grade 1 | 0 | 0 | 5(1.92) | 5 | 1(0.38) | 1 | 6(0.77) | 6 | 0.052 |
| Grade 2 | 0 | 0 | 0 | 0 | 1(0.38) | 1 | 1(0.13) | 1 | >0.999 |
| ≥Grade 3 | 0 | 0 | 0 | 0 | 0 | 0 | 0 | 0 | - |
| **Vomiting** | 0 | 0 | 1(0.38) | 1 | 2(0.77) | 2 | 3(0.38) | 3 | 0.777 |
| Grade 1 | 0 | 0 | 1(0.38) | 1 | 2(0.77) | 2 | 3(0.38) | 3 | 0.777 |
| Grade 2 | 0 | 0 | 0 | 0 | 0 | 0 | 0 | 0 | - |
| ≥Grade 3 | 0 | 0 | 0 | 0 | 0 | 0 | 0 | 0 | - |
| **Loss of appetite** | 0 | 0 | 1(0.38) | 1 | 2(0.77) | 2 | 3(0.38) | 3 | 0.777 |
| Grade 1 | 0 | 0 | 1(0.38) | 1 | 1(0.38) | 1 | 2(0.26) | 2 | >0.999 |
| Grade 2 | 0 | 0 | 0 | 0 | 1(0.38) | 1 | 1(0.13) | 1 | >0.999 |
| ≥Grade 3 | 0 | 0 | 0 | 0 | 0 | 0 | 0 | 0 | - |
| **Hypersensitivity** | 0 | 0 | 0 | 0 | 2(0.77) | 2 | 2(0.26) | 2 | 0.332 |
| Grade 1 | 0 | 0 | 0 | 0 | 1(0.38) | 1 | 1(0.13) | 1 | >0.999 |
| Grade 2 | 0 | 0 | 0 | 0 | 1(0.38) | 1 | 1(0.13) | 1 | >0.999 |
| ≥Grade 3 | 0 | 0 | 0 | 0 | 0 | 0 | 0 | 0 | - |
| **Unsolicited** | 64(24.62) | 89 | 62(23.85) | 85 | 52(20.00) | 73 | 178(22.82) | 247 | 0.406 |
| Grade 1 | 42(16.15) | 54 | 44(16.92) | 60 | 26(10.00) | 39 | 112(14.36) | 153 | 0.048 |
| Grade 2 | 26(10.00) | 31 | 22(8.46) | 25 | 26(10.00) | 32 | 74(9.49) | 88 | 0.788 |
| ≥Grade 3 | 3(1.15) | 4 | 0 | 0 | 1(0.38) | 1 | 4(0.51) | 5 | 0.332 |

N=the number of participant in each group. Grade 1 was a mild reaction, grade 2 was a moderate reaction, and grade 3 was a severe reaction. *p values were generated from the comparison across the three groups, which were calculated using Pearson’s chi-squared test or Fisher’s exact test. When significant difference across vaccine groups was found,we further applied pairwise comparisons on the basis of adjusted ɑ=0.017. **Both the 270ug and 360ug groups showed higher incidences than did the positive control group,but no significant difference was noted between the 270ug and 360ug groups.

**Table S4. List of serious adverse events that occurred throughout study period.**

| Group | ID | SAE term | Dose | Onset time  (days post-vaccination) | Duration  (day) | SAE situation | Relationship to vaccination | Outcome |
| --- | --- | --- | --- | --- | --- | --- | --- | --- |
| 270ug | 0095 | Fracture | 3 | 24 | 6 | Hospitalization | Definitely unrelated | Recovered |
|  | 0396 | Sebaceous cyst excision | 3 | 9 | 3 | Hospitalization | Definitely unrelated | Recovered |
|  | 0513 | Fracture | 2 | 105 | 12 | Hospitalization | Definitely unrelated | Recovered |
|  | 0719 | Smooth muscle cell tumor | 1 | 36 | 5 | Hospitalization | Definitely unrelated | Recovered |
|  | 0719 | Dysfunctional uterine bleeding | 1 | 36 | 5 | Hospitalization | Definitely unrelated | Recovered |
|  | 0746 | Anal fissure | 2 | 88 | 7 | Hospitalization | Definitely unrelated | Recovered |
|  | 0746 | Hemorrhoids | 2 | 88 | 7 | Hospitalization | Definitely unrelated | Recovered |
|  | 0774 | Viral myocarditis | 1 | 48 | 6 | Hospitalization | Definitely unrelated | Recovered |
| 360ug | 0694 | Cervicitis | 1 | 35 | 5 | Hospitalization | Definitely unrelated | Recovered |
|  | 0694 | Cervical polyp | 1 | 35 | 5 | Hospitalization | Definitely unrelated | Recovered |
| positive control | 0458 | Breast cancer | 2 | 6 | 15 | Hospitalization | Definitely unrelated | Recovered |
|  | 0520 | Ruptured ovarian cyst | 3 | 6 | 5 | Hospitalization | Definitely unrelated | Recovered |

SAE: serious adverse event.

**Table S5. The geomentrical mean increases of neutralizing antibody and IgG antibody at month 7 in participants who were baseline seronegative for the corresponding HPV types (PPS).**

| HPV type | 270ug | | 360ug | | positive control | |
| --- | --- | --- | --- | --- | --- | --- |
|  | n/N | GMI  (95%CI) | n/N | GMI  (95%CI) | n/N | GMI  (95%CI) |
| **Neutralizing antibody** | | | | | | |
| HPV6 | 156/156 | 277.03  (244.32,314.12) | 147/147 | 198.90  (174.64,226.52) | 149/149 | 265.55  (232.33,303.52) |
| HPV11 | 230/230 | 82.79  (75.53,90.75) | 223/223 | 71.26  (64.78,78.40) | 217/217 | 135.86  (123.47,149.49) |
| HPV16 | 197/197 | 559.36  (492.43,635.40) | 201/201 | 590.15  (515.14,676.08) | 186/186 | 1218.52  (1073.89,1382.62) |
| HPV18 | 214/214 | 842.09  (726.25,976.41) | 204/204 | 745.04  (655.78,846.44) | 201/201 | 845.79  (736.47,971.33) |
| HPV 31 | 225/225 | 349.97  (311.22,393.54) | 231/231 | 373.26  (330.60,421.42) | 202/226 | 9.17  (7.75,10.86) |
| HPV 33 | 143/143 | 160.42  (140.70,182.90) | 140/140 | 259.51  (225.31,298.90) | 84/138 | 2.14  (1.90,2.42) |
| HPV 45 | 203/203 | 124.07  (110.79,138.93) | 182/182 | 176.39  (155.94,199.51) | 111/181 | 2.11  (1.91,2.33) |
| HPV 52 | 155/155 | 381.74  (326.42,446.43) | 155/155 | 445.49  (387.07,512.73) | 84/144 | 2.19  (1.93,2.50) |
| HPV 58 | 164/164 | 322.68  (282.82,368.17) | 165/165 | 607.51  (528.58,698.22) | 116/166 | 2.90  (2.55,3.30) |
| **IgG antibody** | | | | | | |
| HPV6 | 249/249 | 57.79  (51.85,64.4089) | 249/249 | 49.30  (43.97,55.28) | 242/242 | 46.90  (41.58,52.90) |
| HPV11 | 250/250 | 57.3  (51.23,64.15) | 252/252 | 50.15  (44.43,56.60) | 243/243 | 56.15  (49.52,63.67) |
| HPV16 | 249/249 | 56.41  (50.94,62.46) | 253/253 | 54.51  (49.22,60.37) | 242/242 | 38.98  (34.46,44.08) |
| HPV18 | 251/251 | 61.62  (56.24,67.51) | 252/252 | 59.78  (54.43,65.65) | 244/244 | 38.85  (35.28,42.78) |
| HPV 31 | 250/252 | 42.36  (37.88,47.38) | 253/253 | 43.03  (38.46,48.16) | 138/245 | 4.95  (4.11,5.95) |
| HPV 33 | 250/251 | 54.86  (49.61,60.67) | 250/250 | 55.15  (49.53,61.42) | 142/244 | 5.20  (4.33,6.25) |
| HPV 45 | 251/251 | 42.25  (37.51,45.37) | 255/255 | 41.94  (37.81,46.51) | 158/246 | 6.61  (5.49,7.96) |
| HPV 52 | 251/252 | 51.54  (45.98,57.76) | 255/255 | 52.27  (46.35,58.96) | 144/243 | 5.51  (4.57,6.63) |
| HPV 58 | 249/250 | 47.35  (42.56,52.69) | 253/253 | 51.49  (45.93,57.73) | 136/241 | 4.88  (4.06,5.86) |

PPS=the per-protocol population includes all participants who received all three vaccinations and donated serum samples at day 0 and month 7 within predefined times windows, with no violation of the protocol. GMI=geomentrical mean increases. N=the number of participants in PPS who were baseline seronegative for the corresponding HPV type. n=the number of participants who seroconverted (having 4 times or higher increase of antibody titers) for corresponding HPV type at month 7 in PPS.

**Table S6. Non-inferiority analysis of neutralizing antibody GMT levels at Month 7 in participants who were baseline seronegative for the corresponding HPV types by age groups (PPS)**

| Age group | HPV type | 270ug  (N=254) | | 360ug  (N=256) | | positive control  (N=251) | | GMT ratio(95%CI)  [270ug  /positive control] | GMT ratio(95%CI)  [360ug  /positive control] |
| --- | --- | --- | --- | --- | --- | --- | --- | --- | --- |
|  |  | n | GMT(95%CI) | n | GMT(95%CI) | n | GMT(95%CI) |  |  |
| 20-30 years | HPV6 | 84 | 6958.10  (5931.05,8162.99) | 86 | 4932.49  (4221.99,5762.55) | 89 | 6610.22  (5632.30,7757.94) | 1.05  (0.84,1.32) | 0.75  (0.60,0.93) |
|  | HPV11 | 111 | 1748.42  (1554.90,1966.02) | 106 | 1608.06  (1404.10,1841.65) | 107 | 2924.00  (2536.34,3370.92) | 0.60  (0.50,0.72) | 0.55  (0.45,0.67) |
|  | HPV16 | 103 | 14277.87  (12046.48,16922.58) | 104 | 15923.86  (13330.57,19021.65) | 97 | 31027.44  (26000.67,37026.04) | 0.46  (0.36,0.59) | 0.51  (0.40,0.66) |
|  | HPV18 | 108 | 21447.23  (17735.41,25935.89) | 107 | 18260.64  (15367.13,21698.98) | 104 | 21940.26  (18376.72,26194.84) | 0.98  (0.76,1.27) | 0.83  (0.65,1.06) |
| 31-45 years | HPV6 | 72 | 4247.49  (3534.20,5104.75) | 61 | 2937.47  (2391.98,3607.37) | 60 | 3838.82  (3107.88,4741.67) | 1.11  (0.84,1.46) | 0.77  (0.57,1.02) |
|  | HPV11 | 119 | 1573.89  (1367.50,1811.42) | 117 | 1277.72  (1118.82,1459.18) | 110 | 2530.00  (2223.96,2878.16) | 0.62  (0.51,0.75) | 0.51  (0.42,0.61) |
|  | HPV16 | 94 | 8563.34  (7157.38,10245.47) | 97 | 8561.53  (7077.29,10357.05) | 89 | 18730.61  (15845.92,22140.45) | 0.46  (0.36,0.58) | 0.46  (0.36,0.59) |
|  | HPV18 | 106 | 13165.15  (10556.74,16418.05) | 97 | 11906.86  (9925.79,14283.32) | 97 | 12799.46  (10442.37,15688.59) | 1.03  (0.76,1.39) | 0.93  (0.71,1.22) |

GMT=geometric mean titer. PPS=the per-protocol population includes all participants who received all three vaccinations and donated serum samples at day 0 and month 7 within predefined times windows, with no violation of the protocol. N=the number of participant in each group. n=the number of participant in each age group who were baseline seronegative for neutralizing antibody for the corresponding HPV types.

**Table S7. Superiority analysis of neutralizing antibody GMT levels between groups at Month 7 in participants who were baseline seronegative for the corresponding HPV types by age groups(PPS)**

| Age group | HPV type | 270ug  (N=254) | | 360ug  (N=256) | | positive control  (N=251) | | GMT ratio(95%CI)  [270ug  /positive control] | GMT ratio(95%CI)  [360ug  /positive control] |
| --- | --- | --- | --- | --- | --- | --- | --- | --- | --- |
|  |  | n | GMT(95%CI) | n | GMT(95%CI) | n | GMT(95%CI) |  |  |
| 20-30 years | HPV31 | 116 | 7733.42  (6600.76,9060.44) | 114 | 9631.34  (8155.00,11374.95) | 114 | 249.98  (199.11,313.85) | 30.94  (23.48,40.77) | 38.53  (29.11,51.01) |
|  | HPV33 | 73 | 3687.68  (3096.98,4391.05) | 66 | 5872.75  (4709.65,7323.10) | 67 | 56.21  (47.72,66.21) | 65.61  (51.69,83.27) | 104.48  (79.56,137.21) |
|  | HPV45 | 103 | 2871.54  (2458.77,3353.59) | 93 | 3924.78  (3271.02,4709.20) | 95 | 52.33  (47.02,58.24) | 54.88  (45.50,66.19) | 75.01  (60.78,92.56) |
|  | HPV52 | 80 | 9515.27  (7818.38,11580.45) | 82 | 11190.02  (9429.76,13278.87) | 75 | 59.13  (49.35,70.83) | 160.93  (123.38,209.92) | 189.26  (147.87,242.23) |
|  | HPV58 | 84 | 6280.42  (5189.36,7600.88) | 85 | 13472.63  (10963.46,16556.07) | 87 | 83.77  (71.93,97.57) | 74.97  (58.88,95.46) | 160.82  (124.67,207.47) |
| 31-45 years | HPV31 | 109 | 6294.47  (5287.50,7493.21) | 117 | 5824.15  (4929.99,6880.49) | 112 | 133.89  (105.50,169.92) | 47.01  (35.05,63.07) | 43.50  (32.57,58.10) |
|  | HPV33 | 70 | 2774.69  (2282.93,3372.38) | 74 | 4648.49  (3874.46,5577.15) | 71 | 33.18  (28.25,38.97) | 83.63  (65.11,107.40) | 140.10  (110.03,178.39) |
|  | HPV45 | 100 | 2134.76  (1814.95,2510.93) | 89 | 3155.64  (2674.70,3723.06) | 86 | 33.28  (28.47,38.91) | 64.15  (51.18,80.39) | 94.82  (75.62,118.89) |
|  | HPV52 | 75 | 6036.54  (4749.82,7671.84) | 73 | 6897.69  (5548.20,8575.43) | 69 | 31.74  (27.14,37.11) | 190.20  (143.15,252.71) | 217.33  (166.58,283.55) |
|  | HPV58 | 80 | 6640.64  (5516.06,7994.50) | 80 | 10887.07  (9028.87,13127.67) | 79 | 38.69  (32.57,45.98) | 171.62  (133.46,220.69) | 281.36  (218.55,362.24) |

GMT=geometric mean titer.PPS=the per-protocol population includes all participants who received all three vaccinations and donated serum samples at day 0 and month 7 within predefined times windows,with no violation of the protocol. N=the number of participant in each group. n=the number of participant in each age group who were baseline seronegative for neutralizing antibody for the corresponding HPV types.

**
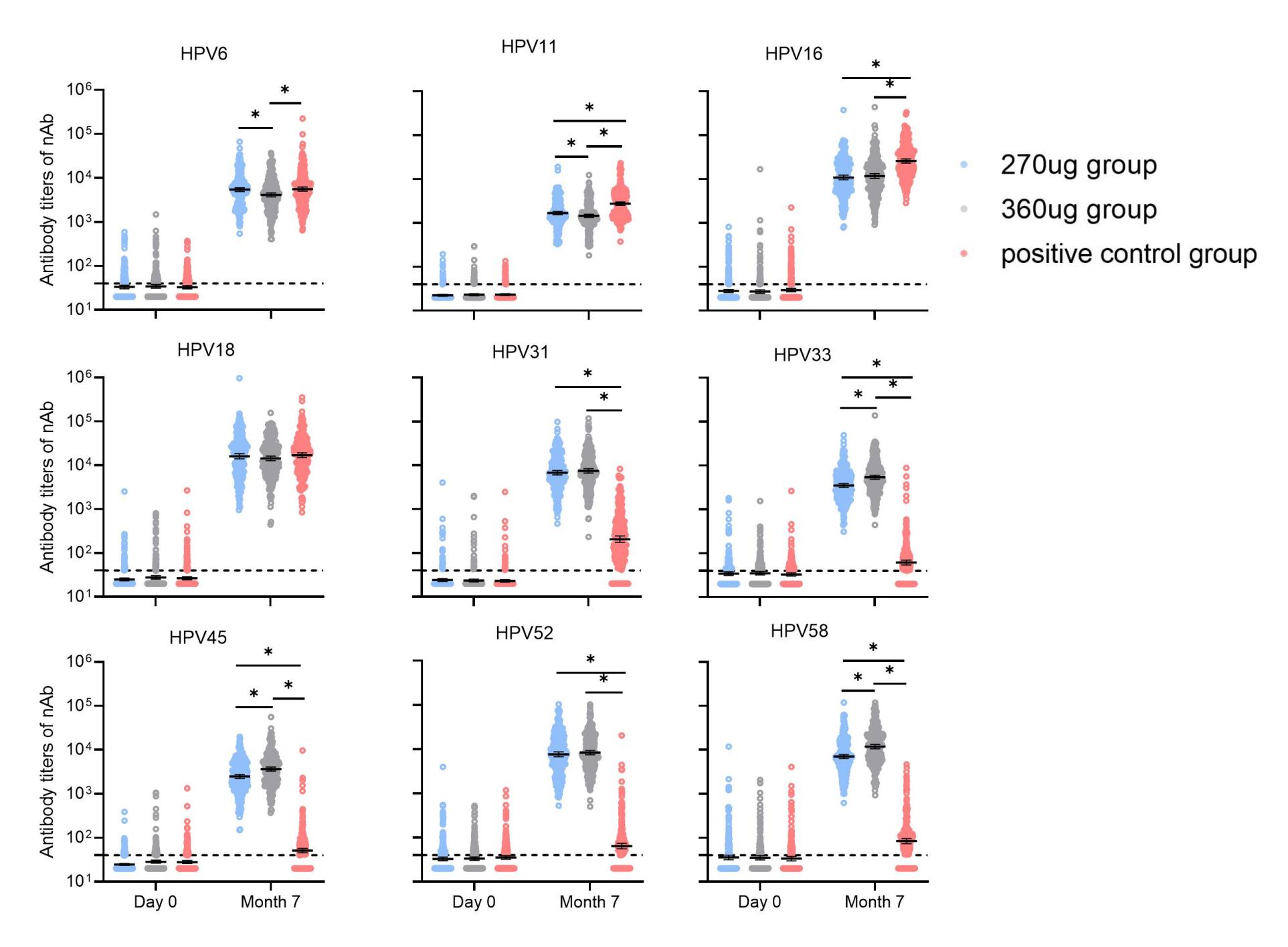
**

**Figure S2. GMTs of neutralizing antibody at day 0 and month 7 in the PPS.** PPS=the per-protocol population includes all participants who received all three vaccinations and donated serum samples at day 0 and month 7 within predefined times windows, with no violation of the protocol. nAb: neutralizing antibody. The dotted lines indicate the cut-off values of neutralizing antibody(40) The black lines indicate the GMT and 95%CI. Antibody titers below the cut-off value were set as half of cut-off for GMT calculation. *: significant difference was found on the basis of adjusted ɑ=0.017.

**
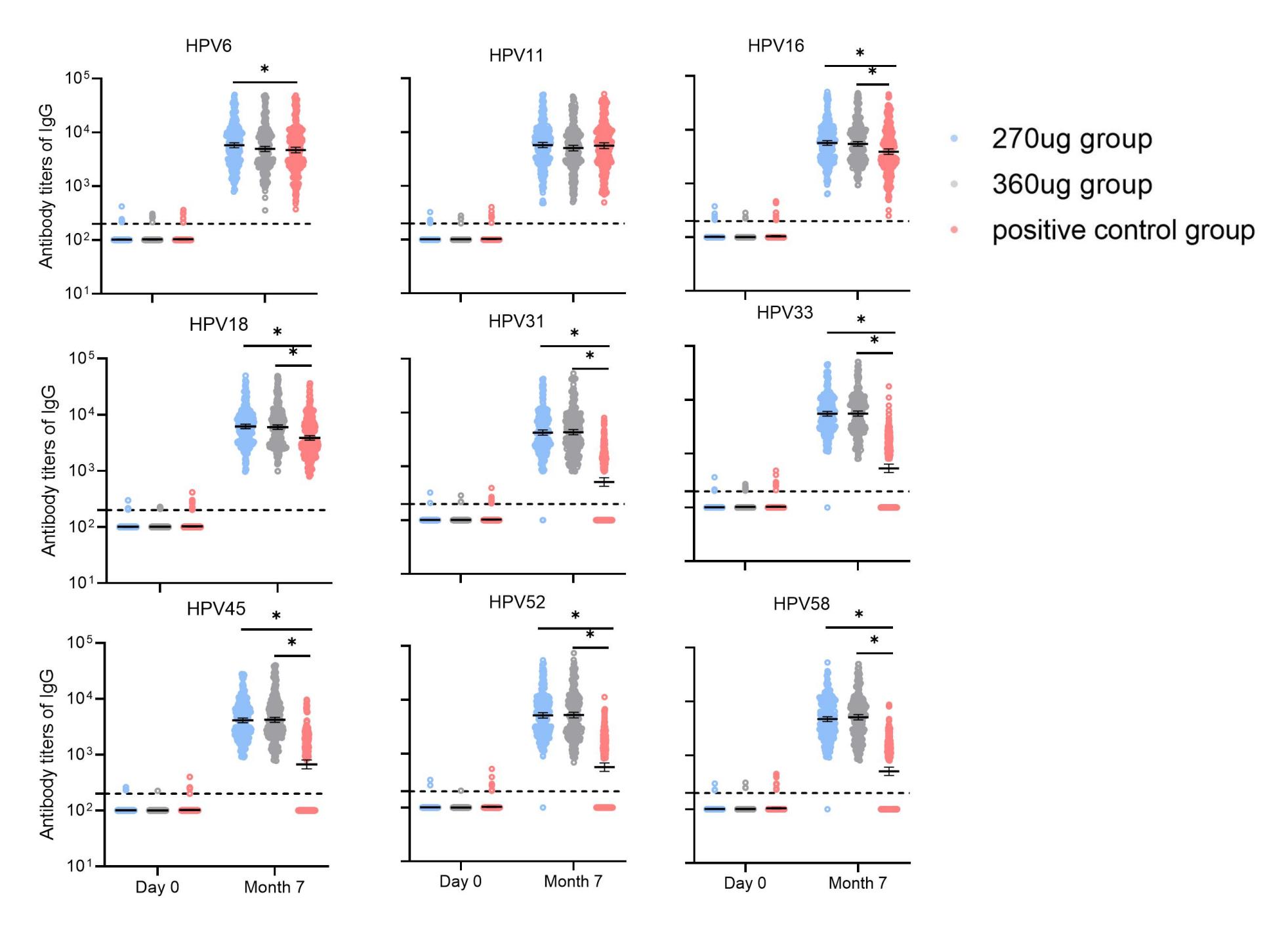
**

**Figure S3. GMTs of IgG antibody at day 0 and month 7 in the PPS.** PPS=the per-protocol population includes all participants who received all three vaccinations and donated serum samples at day 0 and month 7 within predefined times windows, with no violation of the protocol. The dotted lines indicate the cut-off values of IgG antibody(200). The black lines indicate the GMT and 95%CI. Antibody titers below the cut-off value were set as half of cut-off for GMT calculation. *: significant difference was found on the basis of adjusted ɑ=0.017.

**Table S8. Seroconversion rates and geomentrical mean increases of neutralizing antibody and IgG antibody at the month 7 in the PPS.**

| HPV type | 270 ug | | | 360 ug | | | positive control | | |
| --- | --- | --- | --- | --- | --- | --- | --- | --- | --- |
|  | n/N | SCR,%  (95%CI) | GMI  (95%CI) | n/N | SCR,%  (95%CI) | GMI  (95%CI) | n/N | SCR,%  (95%CI) | GMI  (95%CI) |
| **Neutralizing antibody** | | | | | | | | | |
| HPV6 | 254/254 | 100.00 (98.51,100.00) | 163.33  (142.61,187.06) | 256/256 | 100.00 (98.52,100.00) | 121.04  (106.73,137.28) | 251/251 | 100.00 (98.49,100.0) | 170.68  (150.53,193.53) |
| HPV11 | 254/254 | 100.00 (98.51,100.00) | 75.39  (68.55,82.92) | 256/256 | 100.00 (98.52,100.00) | 62.82  (56.96,69.30) | 251/251 | 100.00 (98.49,100.0) | 119.05  (107.90,131.37) |
| HPV16 | 254/254 | 100.00 (98.51,100.00) | 387.89  (333.24,451.50) | 254/256 | 99.22 (97.20,99.79) | 446.34  (388.24,513.14) | 251/251 | 100.00 (98.49,100.0) | 882.82  (769.83,1012.38) |
| HPV18 | 253/254 | 99.61 (97.80,99.93) | 657.24  (562.95,767.32) | 256/256 | 100.00 (98.52,100.00) | 513.67  (440.83,598.55) | 251/251 | 100.00 (98.49,100.0) | 639.81  (550.80,743.20) |
| HPV31 | 252/254 | 99.21 (97.18,99.78) | 290.37  (253.53,332.57) | 256/256 | 100.00 (98.52,100.00) | 314.99  (275.43,360.22) | 218/251 | 86.85 (82.11,90.48) | 12.18  (10.53,14.08) |
| HPV33 | 253/254 | 99.61 (97.80,99.93) | 104.35  (91.96,118.41) | 256/256 | 100.00 (98.52,100.00) | 154.64  (136.29,175.47) | 99/251 | 39.44 (33.60,45.60) | 4.26  (3.64,4.97) |
| HPV45 | 252/254 | 99.21 (97.18,99.78) | 104.35  (93.34,116.67) | 254/256 | 99.22 (97.20,99.79) | 132.48  (116.91,150.12) | 119/251 | 47.41 (41.32,53.58) | 3.61  (3.28,3.97) |
| HPV52 | 253/254 | 99.61 (97.80,99.93) | 238.74  (203.71,279.79) | 255/256 | 99.61 (97.82,99.93) | 255.56  (219.25,297.89) | 96/251 | 38.25 (32.45,44.39) | 4.42  (3.84,5.09) |
| HPV58 | 253/254 | 99.61 (97.80,99.93) | 201.11  (174.58,231.68) | 256/256 | 100.00 (98.52,100.00) | 339.67  (289.31,398.79) | 129/251 | 51.39 (45.24,57.51) | 4.85  (4.39,5.35) |
| **IgG antibody** | | | | | | | | | |
| HPV6 | 254/254 | 100.00 (98.51,100.00) | 56.82  (51.03,63.27) | 256/256 | 100.00 (98.52,100.00) | 48.10  (42.95,53.87) | 251/251 | 100.00 (98.49,100.0) | 45.46  (40.31,51.26) |
| HPV11 | 254/254 | 100.00 (98.51,100.00) | 56.44  (50.46,63.14) | 256/256 | 100.00 (98.52,100.00) | 49.69  (44.07,56.08) | 251/251 | 100.00 (98.49,100.0) | 54.21  (47.86,61.42) |
| HPV16 | 254/254 | 100.00 (98.51,100.00) | 55.58  (50.22,61.51) | 256/256 | 100.00 (98.52,100.00) | 54.08  (48.86,59.86) | 251/251 | 100.00 (98.49,100.0) | 37.56  (33.23,42.44) |
| HPV18 | 254/254 | 100.00 (98.51,100.00) | 61.16  (55.85,66.97) | 256/257 | 100.00 (98.52,100.00) | 59.29  (53.98,65.12) | 251/251 | 100.00 (98.49,100.0) | 37.75  (34.28,41.57) |
| HPV31 | 252/254 | 99.21 (97.18,99.78) | 43.44  (39.14,48.20) | 256/256 | 100.00 (98.52,100.00) | 42.76  (38.25,47.82) | 143/251 | 56.97 (50.79,62.95) | 16.68  (15.27,18.21) |
| HPV33 | 253/254 | 99.61 (97.80,99.93) | 55.08  (50.04,60.62) | 256/256 | 100.00 (98.52,100.00) | 54.24  (48.74,60.36) | 147/251 | 58.57 (52.39,64.49) | 16.48  (15.05,18.05) |
| HPV45 | 254/254 | 100.00 (98.51,100.00) | 40.69  (36.99,44.76) | 256/256 | 100.00 (98.52,100.00) | 41.88  (37.78,46.43) | 161/251 | 64.14 (58.04,69.82) | 18.68  (17.19,20.31) |
| HPV52 | 253/254 | 99.61 (97.80,99.93) | 51.93  (46.52,57.96) | 256/256 | 100.00 (98.52,100.00) | 51.99  (46.10,58.64) | 148/251 | 58.96 (52.79,64.87) | 17.40  (15.96,18.97) |
| HPV58 | 253/254 | 99.61 (97.80,99.93) | 47.26  (42.65,52.37) | 256/256 | 100.00 (98.52,100.00) | 50.57  (45.07,56.74) | 141/251 | 56.18 (49.99,62.17) | 16.25  (14.99,17.62) |

PPS=the per-protocol population includes all participants who received all three vaccinations and donated serum samples at day 0 and month 7 within predefined times windows, with no violation of the protocol. SCR=seroconversion rate. GMI=geomentrical mean increases. N=the number of participants in PPS. n=the number of participants who seroconverted (having 4 times or higher increase of antibody titers) for corresponding HPV type at month 7 in PPS.

**Table S9. Non-inferiority analysis of neutralizing antibody GMTs at month 7 in participants who were IgG baseline seronegative for the corresponding HPV types(PPS).**

| HPV type | 270ug  (N=254) | | 360ug  (N=256) | | positive control  (N=251) | | GMT ratio(95%CI)  [270ug  /positive control] | GMT ratio(95%CI)  [360ug  /positive control] |
| --- | --- | --- | --- | --- | --- | --- | --- | --- |
|  | n | GMT(95%CI) | n | GMT(95%CI) | n | GMT(95%CI) |  |  |
| HPV6 | 249 | 5477.59  (4935.95,6078.65) | 249 | 4126.38  (3713.49,4585.18) | 242 | 5496.01  (4907.78,6154.75) | 0.99  (0.86, 1.16) | 0.75  (0.64, 0.88) |
| HPV11 | 250 | 1690.94  (1547.50,1847.68) | 252 | 1444.70  (1320.90,1580.10) | 243 | 2752.71  (2509.37,3019.63) | 0.61  (0.54, 0.70) | 0.53  (0.46, 0.60) |
| HPV16 | 249 | 10994.40  (9760.65,12384.10) | 253 | 11549.23  (10247.12,13016.79) | 242 | 25716.79  (23018.38,28731.52) | 0.43  (0.36, 0.50) | 0.45  (0.38, 0.53) |
| HPV18 | 251 | 16050.92  (14007.69,18392.18) | 252 | 14289.93  (12655.14,16135.90) | 244 | 17069.31  (15016.61,19402.60) | 0.94  (0.78, 1.13) | 0.84  (0.70, 0.99) |
| HPV31 | 252 | 6840.93  (6100.16,7671.66) | 253 | 7423.76  (6599.34,8351.18) | 245 | 204.38  (172.53,242.10) | 33.47  (27.28, 41.07) | 36.32  (29.55, 44.65) |
| HPV33 | 251 | 3542.77  (3198.51,3924.07) | 250 | 5386.60  (4840.62,5994.16) | 244 | 59.56  (52.70,67.31) | 59.48  (50.72, 69.77) | 90.44  (76.88, 106.40) |
| HPV45 | 251 | 2511.42  (2259.27,2791.72) | 255 | 3637.73  (3272.77,4043.38) | 246 | 51.06  (45.47,57.34) | 49.19  (42.04, 57.55) | 71.24  (60.89, 83.35) |
| HPV52 | 252 | 7701.70  (6785.31,8741.89) | 255 | 8369.93  (7451.10,9402.06) | 243 | 64.88  (55.95,75.22) | 118.71  (97.70, 144.24) | 129.01  (106.88, 155.73) |
| HPV58 | 250 | 6978.31  (6243.02,7800.21) | 253 | 11886.74  (10606.20,13321.89) | 241 | 82.89  (72.29,95.05) | 84.19  (70.57, 100.43) | 143.41  (120.01, 171.37) |

GMT=geometric mean titre. PPS=the per-protocol population includes all participants who received all three vaccinations and donated serum samples at day 0 and month 7 within predefined times windows, with no violation of the protocol. N=the number of participants in each group. n=the number of participants in each group who were baseline seronegative of IgG antibody for the corresponding HPV types.

**Exclusion criteria:**

1. Participants who are pregnant, breastfeeding, or plan to become pregnant within the next 7 months.
2. Participants who received any other HPV vaccine before the first dose of the test vaccine.
3. Participants who used any investigational or unregistered products (drugs or vaccines) within 28 days prior to the first dose of test vaccine, or intend to use such products during the study period.
4. Participants with a history of allergies or known hypersensitivity to any component of the study vaccine (e.g., penicillin, amikacin).
5. Participants with a history of severe adverse reactions to vaccines, including anaphylaxis, urticaria, dyspnea, angioedema, or abdominal pain.
6. Participants with autoimmune diseases, immunodeficiency (including HIV positivity), or primary diseases in vital organs.
7. Asthmatic participants with clinically unstable asthma in the past 2 years requiring emergency treatment, hospitalization, or systemic (oral/intravenous) corticosteroids.
8. Participants with type I or II diabetes (excluding gestational diabetes).
9. Participants with a history of thyroidectomy or thyroid disease requiring treatment within the past 12 months.
10. Participants with severe angioedema within the past 3 years or angioedema requiring medical intervention within the past 2 years.
11. Hypertensive participants with blood pressure >145/95 mmHg despite maintenance pharmacotherapy.
12. Participants with clinically diagnosed coagulation abnormalities (e.g., factor deficiencies, coagulopathies, platelet disorders) or bleeding diatheses.
13. Participants with active malignancy, treated malignancy without documented cure, or high risk of recurrence during the study period.
14. Participants with epilepsy, excluding febrile seizures in children <2 years, alcoholic seizures within 3 years of abstinence, or well-controlled simple epilepsy not requiring treatment for ≥3 years.
15. Participants with asplenia, functional asplenia, or a history of splenectomy for any indication.
16. Participants who received systemic chemotherapy within the past 5 years; or immunosuppressive therapy, cytotoxic treatment, or inhaled corticosteroids (excluding topical corticosteroids for allergic rhinitis or acute non-complicated dermatitis) within the past 6 months.
17. Participants who received blood products within 3 months prior to study vaccine administration.
18. Participants who received live attenuated vaccines within 28 days before the study vaccine.
19. Participants who received subunit or inactivated vaccines (e.g., pneumococcal vaccine) or allergy immunotherapy within 14 days before the study vaccine.
20. Participants undergoing active anti-tuberculosis prophylaxis or treatment.
21. Participants with fever (axillary temperature ≥38.0°C) within 3 days prior to vaccination; or acute illness requiring systemic antibiotic or antiviral therapy within the past 5 days.
22. Participants unable to comply with study protocols due to psychological conditions; or with current or history of psychosis, bipolar disorder (uncontrolled in past 2 years), or psychosis requiring pharmacotherapy; or with suicidal ideation within the past 5 years.
23. Participants deemed ineligible by the investigator due to medical, psychological, social, occupational, or other factors that may compromise study compliance or informed consent.

**IgG antibody**

For the testing of IgG, HPV antigens were diluted using a coating buffer and added to a 96-well plates, which was then coated overnight at 4°C. The coating solution was discarded and plates were washed twice with phosphate-buffered saline (PBS) containing 0.05% Tween-20 and blotted dry. A blocking solution consisting of PBS with 5% skimmed milk powder was added to the wells, and the plate was incubated at room temperature (RT) for 1.5 hours. Following the removal of the blocking solution, the plate was washed once and blotted dry. Serum samples and control samples were then subjected to 4-fold serial dilution using PBS containing 5% skimmed milk powder and added to the plate. After a 1-hour incubation at RT, the liquid was discarded, and the plate was washed 5 times before being blotted dry. Secondary antibody (Anti-Human IgG (Fc specific)-Peroxidase antibody produced in goat, affinity isolated antibody) diluted in PBS with 5% skimmed milk powder was added to the wells. The plate was then incubated at RT for 1 hour, followed by five washes and blotting dry. TMB substrate solution was added, and the color was developed for 15 minutes at RT in the dark. The reaction was stopped by adding a stop solution. The optical density (OD) was measured at a wavelength of 405 nm.

**Neutralizing antibody**

The detection procedure for nAbs was as follows: Approximately 4-8 hours in advance, 293FT cells were seeded into a 96-well plate at a density of 1.5×10⁴ cells per well (100μL). The edge wells were filled with sterile water to prevent evaporation, and the plate was incubated at 37°C with 5% CO₂. Test sera were heat-inactivated in a 56°C water bath for 30 minutes. Pre-immunization sera were initially diluted 40-fold in a 96-well deep-well plate, followed by 4-fold serial dilutions. Post-immunization sera underwent the same initial dilution but were serially diluted 8-fold (up to 1:655360). For BPV testing, sera were initially diluted 10-fold and then serially diluted across 3 gradients. All dilution steps were performed using a multichannel pipette with thorough mixing. The diluted sera were transferred to a 96-well U-bottom plate (with replicate wells), mixed with pseudovirus diluted to ≥200 plaque-forming units, and incubated at 4°C for 1 hour. Subsequently, 100 μL of the mixture was transferred to the pre-seeded 96-well cell plate and cultured at 37°C with 5% CO₂ for 60-96 hours. Detection was performed using an ELISPOT reader. The percent infection inhibition was calculated using the formula: Percent Inhibition = [1 - (Sample value - Cell control value) /(Virus control value - Cell control value)] × 100%. The ID₅₀ was defined as the highest serum dilution that yielded an inhibition greater than 50%. Assays required retesting if the virus control value was <200, the replicate well variation exceeded 30%, results showed an inverted pattern, or the calculated titer exceeded the maximum dilution tested.

**GST and 3C protease antibody**

For the testing of GST, firstly, dilute the concentrated wash buffer 20-fold with distilled water, perform serial two-fold dilution of the standard with sample dilution buffer, prepare the working solution of enzyme-labeled antibody freshly according to the instruction manual, and mix solution A and solution B at a ratio of 1:1 in the dark before using the substrate solution. Subsequently, dilute the GST antigen to 2 μg/ml with coating buffer, add it to the 96-well plate, and coat at 4℃ for 12-18 hours. After discarding the coating solution, wash the plate twice with the diluted wash buffer and pat it dry, then add the dilution containing 5% skimmed milk powder and block at room temperature (RT) for 2 hours. Discard the blocking solution, wash the plate once and pat it dry, add the prepared control serum and test serum, incubate at RT for 1 hour, then discard the liquid in the wells and wash the plate repeatedly 5 times with the wash buffer before patting it dry. Next, add the enzyme-labeled working solution and incubate at RT for 1 hour. After discarding the reaction solution, wash the plate 5 times again with the wash buffer and pat it dry. Add the chromogenic substrate solution and incubate at RT in the dark for 5 minutes. Then add the stop solution to terminate the reaction, and place the plate in a microplate reader to read the absorbance at a wavelength of 450 nm. The detection for 3C protease antibody is identical to that for GST antibody except for the coating antigen, all other steps are exactly the same.
